# Neogene sharks and rays from the Brazilian ‘Blue Amazon’

**DOI:** 10.1371/journal.pone.0182740

**Published:** 2017-08-23

**Authors:** Orangel Aguilera, Zoneibe Luz, Jorge D. Carrillo-Briceño, László Kocsis, Torsten W. Vennemann, Peter Mann de Toledo, Afonso Nogueira, Kamilla Borges Amorim, Heloísa Moraes-Santos, Marcia Reis Polck, Maria de Lourdes Ruivo, Ana Paula Linhares, Cassiano Monteiro-Neto

**Affiliations:** 1 Departamento de Biologia Marinha, Instituto de Biologia, Universidade Federal Fluminense, Niterói, Rio de Janeiro, Brasil; 2 Instituto de Geociências, Universidade Federal do Pará, Belém, Pará, Brasil; 3 Palaeontological Institute and Museum, University of Zürich, Zürich, Canton of Zürich, Switzerland; 4 Faculty of Science, Geology Group, University of Brunei Darussalam, Jalan Tungku, Gadong, Brunei Darussalam; 5 Institut des Dynamiques de la Surface Terrestre, Université de Lausanne, Lausanne, Vaud, Switzerland; 6 Instituto Nacional de Pesquisas Espaciais, São José dos Campos, São Paulo, Brasil; 7 Instituto de Astronomia, Geofísica e Ciências Atmosféricas, Universidade de São Paulo, São Paulo, Brasil; 8 Coordenação de Ciências da Terra e Ecologia, Museu Paraense Emilio Goeldi, Belém, Pará, Brasil; 9 Departamento Nacional de Produção Mineral, Rio de Janeiro, Rio de Janeiro, Brasil; University of Alabama, UNITED STATES

## Abstract

The lower Miocene Pirabas Formation in the North of Brazil was deposited under influence of the proto-Amazon River and is characterized by large changes in the ecological niches from the early Miocene onwards. To evaluate these ecological changes, the elasmobranch fauna of the fully marine, carbonate-rich beds was investigated. A diverse fauna with 24 taxa of sharks and rays was identified with the dominant groups being carcharhiniforms and myliobatiforms. This faunal composition is similar to other early Miocene assemblages from the proto-Carribbean bioprovince. However, the Pirabas Formation has unique features compared to the other localities; being the only Neogene fossil fish assemblage described from the Atlantic coast of Tropical Americas. Phosphate oxygen isotope composition of elasmobranch teeth served as proxies for paleotemperatures and paleoecology. The data are compatible with a predominantly tropical marine setting with recognized inshore and offshore habitats with some probable depth preferences (e.g., *Aetomylaeus* groups). Paleohabitat of taxa particularly found in the Neogene of the Americas (†*Carcharhinus ackermannii*, †*Aetomylaeus cubensis*) are estimated to have been principally coastal and shallow waters. Larger variation among the few analyzed modern selachians reflects a larger range for the isotopic composition of recent seawater compared to the early Miocene. This probably links to an increased influence of the Amazon River in the coastal regions during the Holocene.

## Introduction

**Fig 1:**
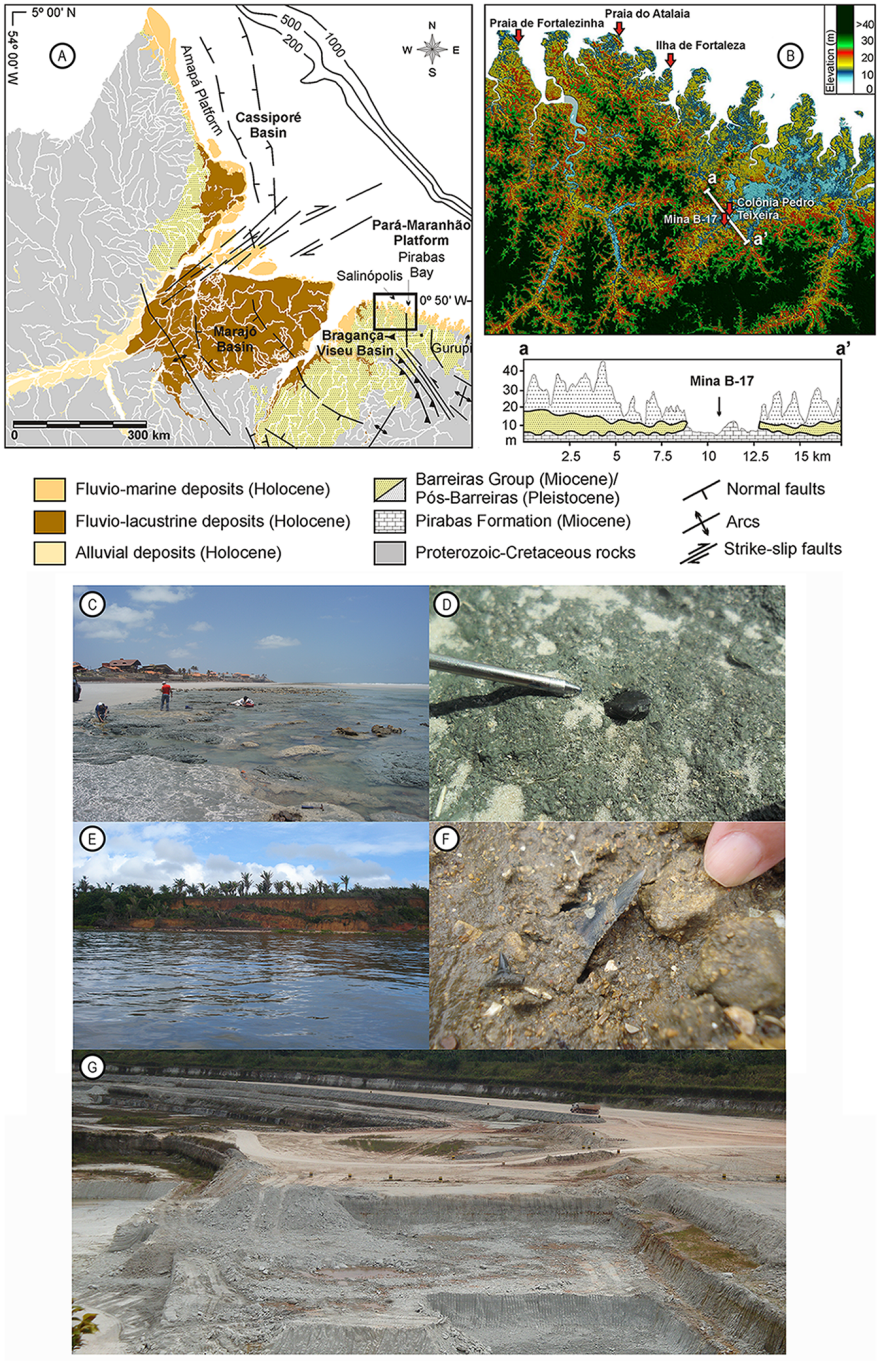
Location map. **A**. Regional geology, **B**. Digital elevation model of the northern coast of the Pará state (modified from Aguilera et al. [7]). **C**, **D**. Atalaia outcrop and detail showing fossil shark teeth. **E, F**. Fortalezinha Island outcrop and detail showing fossil shark teeth. **G**. Capanema Mine B-17 partial view.

**Fig 2:**
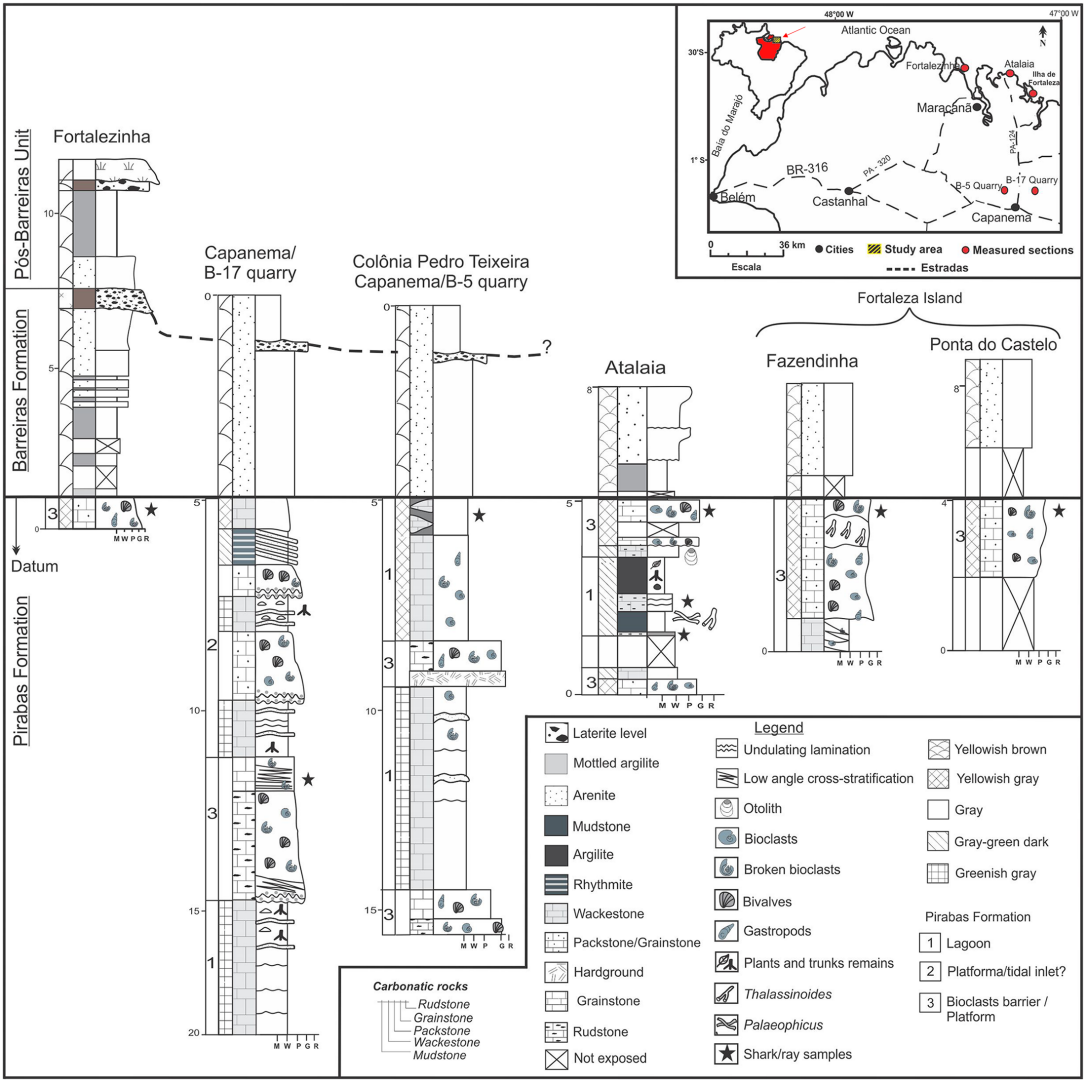
Composite sections of Pirabas, Barreiras and Pós-Barreira formations.

The evolution of the Amazon River and its drainage basin are closely related to the uplift of the Andes at the northwestern coast of South America [1–3]. During the early Miocene the influence of the river was not as important as it is today and many tropical Neogene marine basins existed at the northern coast of Brazil. The sediments deposited onto the Precambrian rocks at the coastal margin of the Guyana and the Brazilian shields [4,5] are mainly biogenic carbonates and siliciclastic rocks with an exceptional abundance and diversity of a shallow marine fossil fauna [6,7]. These sedimentary sequences are linked to global sea-level variations, and two regionally transgressive episodes may be distinguished along the Brazilian coast: one in the Oligo-Miocene and another in the early to middle Miocene [5,8,9]. Here the early Miocene (Aquitanian-Burdigalian) carbonate unit of the Pirabas Formation [5,10] is studied from the Eastern Graben of Marajó in the Bragantina Platform, in northern Brazil (Fig 1). During the Cenozoic similar shallow water carbonates were common [11], however, in Brazil these platforms had a different evolution mainly in the Equatorial Atlantic basins. In the Bragantina Platform carbonates were gradually replaced by siliciclastic sediments of the Barreiras Formation, which represent the expressive progradation of continental deposits linked to the last thermal tectonic event in the North to Eastern Brazilian coastal margin during the middle to late Miocene (Fig 2). In contrast, the carbonate platforms in the Western Brazilian Coast, clearly indicate the step-wise hydrographic changes related to the enlargement of the main Amazon drainage system since the Pliocene-Pleistocene (e. g., [3,5,12–18]). This event also triggered changes in the coastal marine environment together with sea level variation through time [19].

The investigated unit is the Pirabas Formation, deposited during a relative global warm period [20] that preceded the middle Miocene Climatic Optimum [21]. Its fauna rich in species and abundance is well known [10,22] and a distinct tropical Western Central Atlantic subprovince was proposed for the early Miocene, based on the benthic marine invertebrates (mollusks, crustaceans, echinoids, corals and bryozoans). The formation also yielded many fish remains, among them elasmobranchs [23–26], and most recently a very diverse shallow marine and brackish teleostean fauna was reported [6,27,28].

This study examines the elasmobranch remains (shark and ray teeth) of the fully marine series of the Pirabas Formation. First, the taxonomy of the recovered fossils is considered, and then selected, well-preserved shark and batoid teeth were chosen for stable isotope analyses (δ^18^O_PO4_, δ^18^O_CO3_, δ^13^C) for paleoenvironmental interpretation. The phosphate oxygen isotope composition of shark teeth is often used as a proxy for describing environmental and ecological conditions for both extant and fossil taxa [29–38]. This is due to the fact that shark teeth are biominerals with enameloid that is primarily composed of fluorapatite [39,40], the least soluble apatite and most resistant to subsequent alteration [41]. In contrast, batoids only have a single, thin layer of enameloid and most of their crown is comprised of dentine [42–44]. However, their δ^18^O_PO4_ values still can provide useful paleoecological information if the data are carefully interpreted, especially when other geochemical methods are used in parallel to help constrain post-mineralization alteration [29,30,45–49]. One such proxy is the stable isotope composition of the structural carbonate (δ^18^O_CO3_, δ^13^C) that can help trace diagenetic alteration (e. g., proportion of dentine in the sample), or if unaltered may provide information about the sources of carbon in the paleoecosystem and/or in the depositional environment. So far only a few δ^18^O_PO4_ analyses of chondrichthyan bioapatite have been measured from South America, largely from the Pacific coast: the middle Miocene-early Pliocene Pisco Formation in Peru and the Plio-Pleistocene Canoa Formation in Ecuador [50,51].

Here a multidisciplinary approach is used to help understand the paleoecological aspects of Amazonian elasmobranchs and complement knowledge on the Pirabas paleoenvironment within a wider geographic context. The isotope data of the early Miocene aquatic fauna will be discussed in view of likely regional adaptative events as a consequence of the prograding Plio-Pleistocene Amazon and Orinoco deltas.

## Material and methods

The outcrops of the Pirabas Formation [10,52] were explored along coastal cliffs during low tide in the “Salgado region”, State of Pará and in the open pit quarries near Capanema city (Fig 1). Field trips to the type locality of the Pirabas Formation were conducted in Ilha de Fortaleza, São João de Pirabas Municipality (0° 37’ 33” S, 47° 32’ 30” W), and in the Ilha de Fortalezinha, Maracanã Municipality (0° 37’ 33” S, 47° 32’ 30” W), Colônia Pedro Teixeira, Capanema Municipality (1° 10’ 38” S, 47° 13’ 00” W), B-17 quarry of CIBRASA, Capanema Municipality (1° 2’ 47” S, 47° 9’ 26” W) and Praia de Atalaia outcrop, Salinopolis Municipality (0° 35’ 37” S, 47°18’ 54.4” W), State of Pará, Brazil, where the main stratigraphic sections were measured (Figs 1 and 2).

Large specimens were collected directly from the outcrops, following the classical stratigraphic successions of the Pirabas Formation presented previously in several works [6,19,27,53–57]. In addition, 30 kg of sediments were collected in the Atalaia section, screen-washed and sieved with 0.5, 1.0 and 2.0 mm open mesh-size, dried and picked under a stereomicroscope to examine the presence and relative abundance of microdental elements.

The fossiliferous localities of Sitio da Olaria, Sitio Pedro Teixeira and B-11 and B-5 quarries (Capanema Municipality) were destroyed by industrial mining activity, agriculture and urban development. As a consequence, only the specimens collected in the 1940s and 1950s were studied from the collections at the Museu de Ciências da Terra from the Companhia de Pesquisa de Recursos Minerais (CPRM) and in the Museu Nacional at Universidade Federal do Rio de Janeiro (MN UFRJ). All necessary permits for fieldwork, laboratory analyzes and descriptions conducted by the team from the Museum Paraense Emilio Goeldi and the Universidade Federal do Pará were provided by the Departamento Nacional de Produção Mineral (DNPM), which complied with all relevant regulations.

All specimens collected during this project are housed in the paleontological collection of Museu Paraense Emilio Goeldi (MPEG-V), Brazil. Specimen numbers are provided in the supplementary appendix with repository information of studied species (S1 Appendix). All specimens from the studies of Santos and Travassos [23], Reis [25], and Costa et al. [26], were reviewed and included in our study. Elasmobranch taxonomic classification follows Compagno [58,59] and Cappetta [42]; terminology is based on Cappetta [42]. Taxonomic identifications are based on an extensive literature review (e.g. [23,25,26,42,60–83]) and comparative analyses between fossil and extant specimens from the following collections: Departamento Nacional de Pesquisas Minerais (DNPM), Brazil; Museu Paraense Emilio Goeldi (MPEG-V), Brazil; Natural History Museum of Basel (NMB), Switzerland; Paleontological collections of the Alcaldía de Urumaco (AMU-CURS), Venezuela; Palaeontological Institute and Museum at the University of Zurich (PIMUZ) Switzerland; René Kindlimann (private collection), Switzerland.

52 selected fossil teeth of 10 selachian taxa were used for isotope analyses (δ^18^O_PO4_). The taxa and their isotopic values are shown in Table 1. To complement the study, fossil shark teeth (10 specimens of †*H*. *serra*) from proto-Caribbean Neogene deposits were also analyzed, serving as an additional comparative basis of prevalent tropical settings [50,51].

**Table 1:** Fossil elasmobranch specimens used in geochemical investigation.

Sample ID	Taxon	Locality	δ^18^O_PO4_	δ^18^O_PO4_	Derived T (°C)
			(VSMOW)	Standar Desv.	
GL-I	†*Galeocerdo mayumbensis*	B-5 Mine	19.7	0.1	26.6
GL-II	†*Galeocerdo mayumbensis*	B-17 Mine	18.9	0.1	30.1
GL-III	†*Galeocerdo mayumbensis*	B-17 Mine	19.8	0.1	26
GL-IV	†*Galeocerdo mayumbensis*	B-17 Mine	19.7	0.1	26.4
GL-V	†*Galeocerdo mayumbensis*	B-17 Mine	19.8	0.1	25.9
GL-VI	†*Galeocerdo mayumbensis*	B-17 Mine	19.8	0.1	26
HS-I	†*Hemipristis serra*	B-17 Mine	19.3	0.1	28.2
HS-II	†*Hemipristis serra*	B-17 Mine	19.5	0.1	27.5
HS-III	†*Hemipristis serra*	B-17 Mine	19.6	0.2	26.9
HS-IV	†*Hemipristis serra*	Atalaia outcrop	19.9	0.1	25.6
HS-V	†*Hemipristis serra*	Atalaia outcrop	19.7	0.1	26.7
HS-VI	†*Hemipristis serra*	Atalaia outcrop	19.8	0.2	26
CP-I	*Carcharhinus* sp.	B-5 Mine	19.3	0.1	28.1
CP-II	*Carcharhinus* sp.	B-5 Mine	19.7	0.3	26.3
CP-III	*Carcharhinus* sp.	D-11 Mine	18.9	0	30
CP-IV	*Carcharhinus* sp.	B-17 Mine	19.2	0.4	28.8
CA-I	†*Carcharhinus ackermannii*	B-17 Mine	19.1	0	29.1
CA-II	†*Carcharhinus ackermannii*	B-17 Mine	19.1	0.2	29.1
CA-III	†*Carcharhinus ackermannii*	B-17 Mine	19.4	0.3	27.8
CA-IV	†*Carcharhinus ackermannii*	B-17 Mine	19.6	0.2	26.7
SM-I	*Sphyrna* sp.	B-17 Mine	20.3	0.1	23.9
SM-II	*Sphyrna* sp.	B-17 Mine	19.7	0.2	26.7
SM-III	*Sphyrna* sp.	B-17 Mine	19.6	0.1	26.8
SM-IV	*Sphyrna* sp.	B-17 Mine	20	0	25.3
SM-V	*Sphyrna* sp.	B-17 Mine	19.2	0.3	28.8
SM-VI	*Sphyrna* sp.	B-17 Mine	19.1	0.1	29.2
CB-I	†*Carcharocles chubutensis*	B-17 Mine	20.1	1 sub-sample	24.5
CB-II	†*Carcharocles chubutensis*	Atalaia outcrop	20.3	0.2	23.8
CB-III	†*Carcharocles chubutensis*	B-5 Mine	19.4	0.1	27.8
CB-IV	†*Carcharocles chubutensis*	B-5 Mine	19.5	0.1	27.4
CB-V	†*Carcharocles chubutensis*	B-5 Mine	19.9	0.4	25.5
AC—I	†*Aetomylaeus cubensis*	Atalaia outcrop	19.3	0.2	28.2
AC—II	†*Aetomylaeus cubensis*	Atalaia outcrop	19.4	0.3	27.7
AC—III	†*Aetomylaeus cubensis*	Atalaia outcrop	19.6	0	27.1
AC—IV	†*Aetomylaeus cubensis*	B-17 Mine	19.9	0.2	25.6
AE—I	*Aetomylaeus* sp.	Atalaia outcrop	20.4	0.2	23.3
AE—II	*Aetomylaeus* sp.	Atalaia outcrop	20	0.3	25.3
AE—III	*Aetomylaeus* sp.	Atalaia outcrop	20	0	25.3
AE—IV	*Aetomylaeus* sp.	Atalaia outcrop	20.1	0.1	24.9
AE—V	*Aetomylaeus* sp.	Atalaia outcrop	20	0.1	25.4
AE—VI	*Aetomylaeus* sp.	Atalaia outcrop	19.5	0.3	27.5
AE—VII	*Aetomylaeus* sp.	Atalaia outcrop	19.7	0.1	26.5
AE—VIII	*Aetomylaeus* sp.	Atalaia outcrop	20.1	0.1	24.6
AE—IX	*Aetomylaeus* sp.	Atalaia outcrop	20.1	0.3	24.8
RH—I	*Rhinoptera* sp.	Atalaia outcrop	19.8	0.2	26.2
RH—II	*Rhinoptera* sp.	Fortalezinha outcrop	20.4	0	23.1
MY—I	Myliobatoidea	Fortalezinha outcrop	20.1	0,2	24.8
MY—II	Myliobatoidea	Fortalezinha outcrop	19.5	0.1	27.3
MY—III	Myliobatoidea	Atalaia outcrop	20.5	0	23.1
MY—IV	Myliobatoidea	Atalaia outcrop	19.3	0.2	28.4
MY—V	Myliobatoidea	Atalaia outcrop	19.5	0	27.5
PT—I	*Pristis* sp.	Atalaia outcrop	19.4	0.3	27.9
HS-VII	†*Hemipristis serra*	Cantaure Fm (Venezuela)	20.8	0.1	21.7
HS-VIII	†*Hemipristis serra*	Cantaure Fm (Venezuela)	20.0	0.0	24.9
HS-IX	†*Hemipristis serra*	Cantaure Fm (Venezuela)	19.8	0.2	26.2
HS-X	†*Hemipristis serra*	Caujaurao Fm (Venezuela)	20.6	0.1	24.6
HS-XI	†*Hemipristis serra*	Jimol Fm (Colombia)	20.1	0.1	24.9
HS-XII	†*Hemipristis serra*	Jimol Fm (Colombia)	20.0	0.0	25.2
HS-XIII	†*Hemipristis serra*	Jimol Fm (Colombia)	19.8	0.1	25.8
HS-XIV	†*Hemipristis serra*	Jimol Fm (Colombia)	19.9	0.1	25.8
HS-XV	†*Hemipristis serra*	Castilletes Fm (Colombia)	19.8	0.2	26.2
HS-XVI	*†Hemipristis serra*	Chagres Fm (Panama)	20.6	0.2	24.7

Oxygen isotopic composition of elasmobranch teeth from the Pirabas Formation and fossil shark teeth from complementary Neogene deposits (n = 62).

Teeth (n = 10) of the modern bullshark *Carcharhinus leucas* Müller and Henle 1839 [84] from the inner shelf of the Bragantina coast in the Pará state, were also analyzed (Table 2). This species was selected due to its known long-term migratory habitat into estuarine river systems [85] and hence can be compared to the Amazonian fossils in terms of freshwater influence on marine waters.

**Table 2:** Extant sharks used in geochemical investigation.

Sample ID	Taxon	Locality	δ^18^O_PO4_ (VSMOW)	δ^18^O_PO4_ Standar Desv.	Derived T (°C)
CL-Ia	*Carcharhinus leucas*	Amazon delta	20.2	0.1	28.9
CL-Ib	*Carcharhinus leucas*	Amazon delta	20.3	0.1	28.4
CL-Ic	*Carcharhinus leucas*	Amazon delta	20.7	0,2	26.5
CL-1d	*Carcharhinus leucas*	Amazon delta	20.9	0.1	25.6
CL-1e	*Carcharhinus leucas*	Amazon delta	20.9	0.1	25.7
CL-1f	*Carcharhinus leucas*	Amazon delta	20.8	0.1	26
CL-II	*Carcharhinus leucas*	Amazon delta	20.9	0.1	25.6
CL-III	*Carcharhinus leucas*	Amazon delta	21.4	0.2	23.6
CL-IV	*Carcharhinus leucas*	Amazon delta	19.6	0.1	31.5
CL—V	*Carcharhinus* sp.	Amazon delta	19.9	0.1	30

Oxygen isotopic composition of extant shark from the Amazon Delta region (n = 10).

Stable isotope analyses of the shark and ray teeth (n = 72, Tab. 1, 2) were done at the Stable Isotope Laboratory of the University of Lausanne, Switzerland. The focus was on the more resistant phosphate derived δ^18^O_PO4_, however, the isotopic composition of the structural carbonate in apatite (δ^18^O_CO3_ & δ^13^C) was also analyzed. All teeth were cleaned in Milli-Q water in an ultrasonic bath to reduce sedimentary contamination. Preferentially shark tooth enameloid was sampled, but some amount of dentine could have remained in some fossil samples where the tip (apex of crown) of the small teeth was taken. The relative proportion of dentine was cross-checked by the analyses derived from the structural carbonate measurements, given that dentine has a higher carbonate content and lower carbon isotopic composition compared to enameloid [29,37]. In previous studies δ^18^O_PO4_ from enameloid and dentine showed no significant differences for the given setting [29,37]. Moreover, bulk sampling is commonly employed when only small teeth or not enough samples are available [33–35,86]. In the case of batoids, most of the sampled material consists of dentine. Sample procedures and analyses are summarized in S2 Appendix.

## Geological setting (Pirabas Formation)

The stratigraphic sections (Fig 2) are characterized by massive mudstone with trace fossils, bioturbation, plant remains, pyrite concretions, and massive to laminated wackestones with plant fragments. The wackestones and packstones/grainstones have low angle of cross-stratification; the hardground is rich in bryozoans, rudstones and contain broken or well-preserved invertebrate fossils. These facies and micro-facies were interpreted in the general context of representing marginal lagoon/mangrove, tidal inlets, and bioclastic bars/platform paleoenvironments.

The marginal lagoon/mangrove consists of mudstone with pyrite concretions and plant remains with a thickness of about 80 cm (more restricted occurrence). *Thalassinoides* and *Gyrolithes* ichnofossils were found in the mudstone layer in a thickness level of about 50 cm. Massive wackstone with wavy laminations yielded both well-preserved and broken invertebrate fossils. The thickness of these layers ranged between 2 to 5 m. These facies associations were deposited in a paleoenvironment with low energy deposition by suspension in the limit between the oxic-anoxic zones, in agreement with the presence of pyrite.

The tidal inlet deposits are characterized by recurrent bioturbation in the first meter, wackestone with wavy lamination with up to 2 m; the fossiliferous packstones/grainstones has whole and fragmented invertebrate fossils arranged in recurrent beds with up to 2.5 m of thickness. This facies association represents moderate to high energy channels, dominated by ebb and flood tidal currents that were reworked continuously, sporadically sands were transported by currents and deposition by suspension occurred during low level stands.

The bioclastic bars/platform deposits are characterized by 70 cm-thick low angle cross-stratified wackestones. Fossiliferous packstones/grainstones contain fragments or entire invertebrates in layers with up to 4 m of thickness. Grainstones and hardgrounds with 50 cm thickness exhibit abundant bryozoans and the rudstone beds, with up to 3 m thick, exhibit well-preserved invertebrate fossils. This facies association represents moderate to high energy setting frequently reworked by oscillatory flow.

## Results

### Fish assemblage

**Fig 3:**
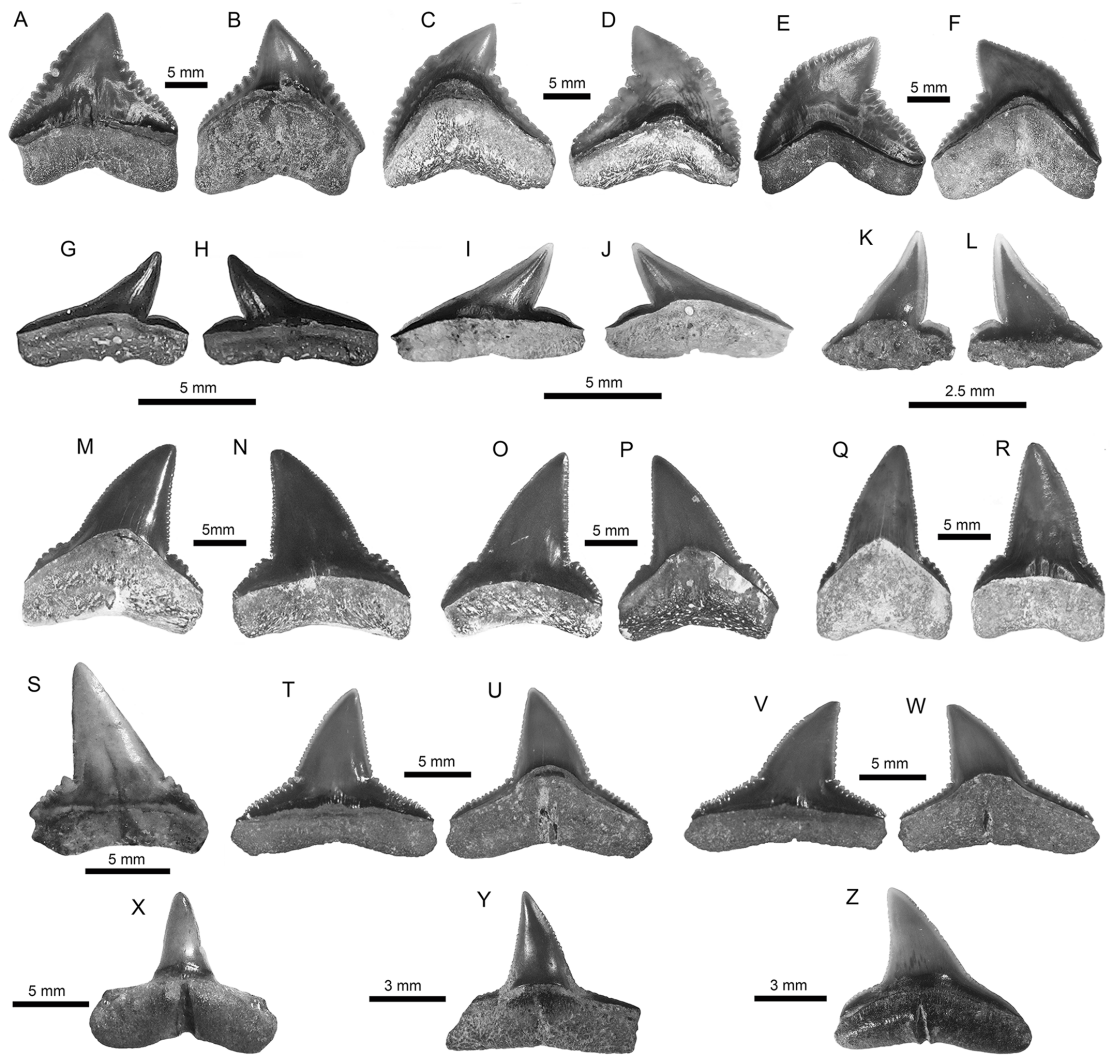
Carcharhiniformes of the Pirabas Formation. A-F. †*Galeocerdo mayumbensis* (A-B: MPEG-1710-V; C-D: MPEG-177-V; E-F: MPEG-1854-V). G-L. *Rhizoprionodon* sp. (G-H: MPEG-1707-V; I-J: MPEG-1708-V; K-L: MPEG-1837-V). M-R. †*Carcharhinus ackermannii* (M-N: MPEG-1131-V; O-P: MPEG-1133-V; Q-R: MPEG-824-V). S. †*Carcharhinus gibbesii* (MGM-DNPM-969-P). T-W. *Carcharhinus perezi* (T-W: MPEG-1836-V). X-Z. *Carcharhinus* sp. (X: MPEG-842-V; Y: MPEG-1928-V; Z: MPEG-1927-V). View: labial (A, D-E, H-I, L, N-O, R-T, V), lingual (B-C, F-G, J-K, M, P-Q, V, W-Z).

**Fig 4:**
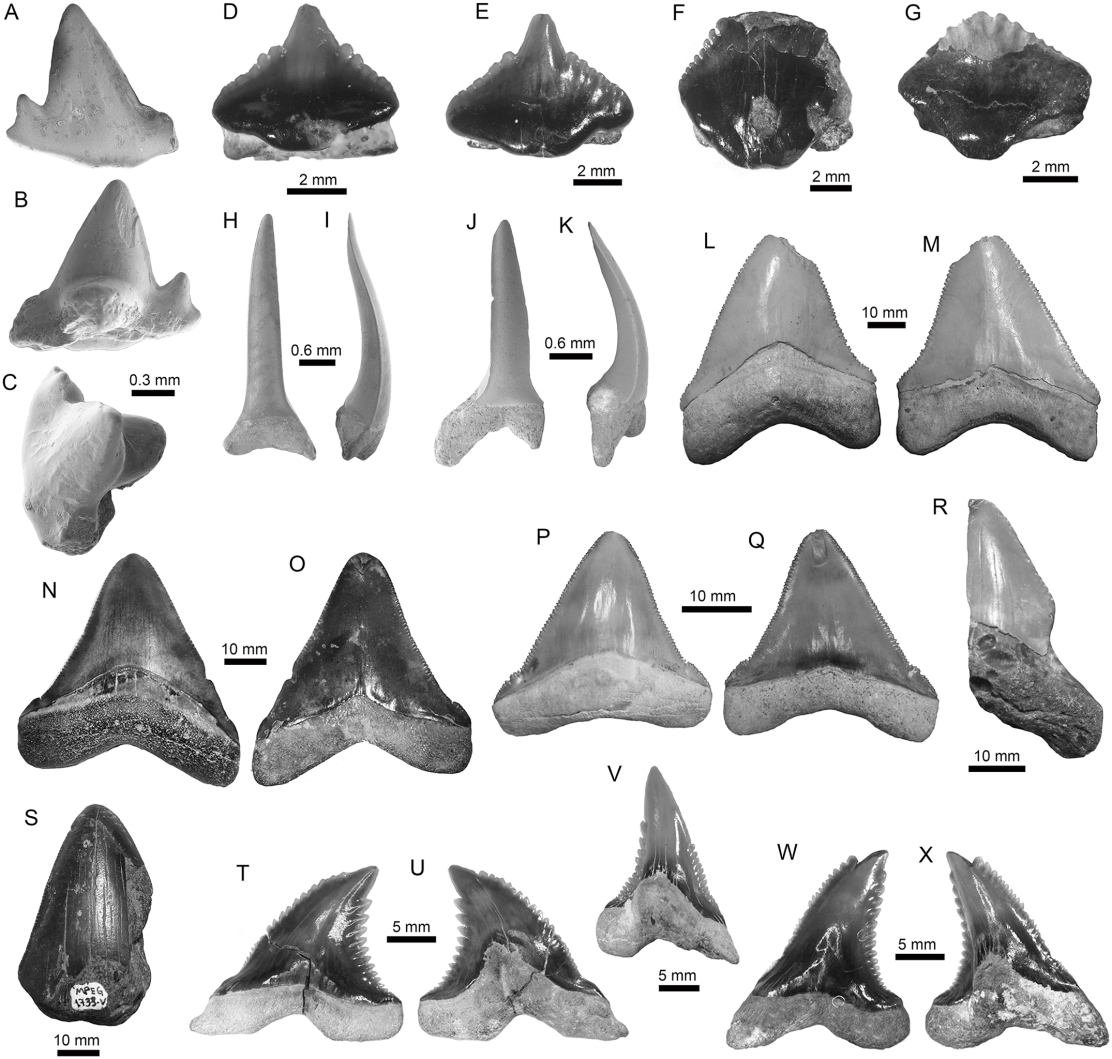
Orectolobiformes, Lamniformes and Carcharhiniformes of the Pirabas Formation. A-C. cf. *Chiloscyllium* sp. (MPEG-1956-V); D-G. *Nebriu*s sp. (D: MPEG-1073-V; E: MPEG-1546-V; F: MPEG-1545-V; G: MPEG-814-V). H-K. *Pseudocarcharias* cf. *P*. *komoharai* (H-I: MPEG-1852-V; J-K: MPEG-1851-V). L-Q. †*Carcharocles chubutensis* (L-M: MGM-DNPM-967-P; N-O: MPEG-723-V; O-P: MPEG-1733-V). R-S. †*Carcharocles* sp. (R: MPEG-97-V; S: MPEG-1733-V). T-X. †*Hemipristis serra* (T-U: MPEG-781-V; V: MPEG-725-V; W-X: MPEG-727-V). View: labial (A, D, G-H, J, M, O, Q, T, W), lingual (B, L, N, P, R-S, U-V, X), profile (I, K), occlusal (C).

**Fig 5:**
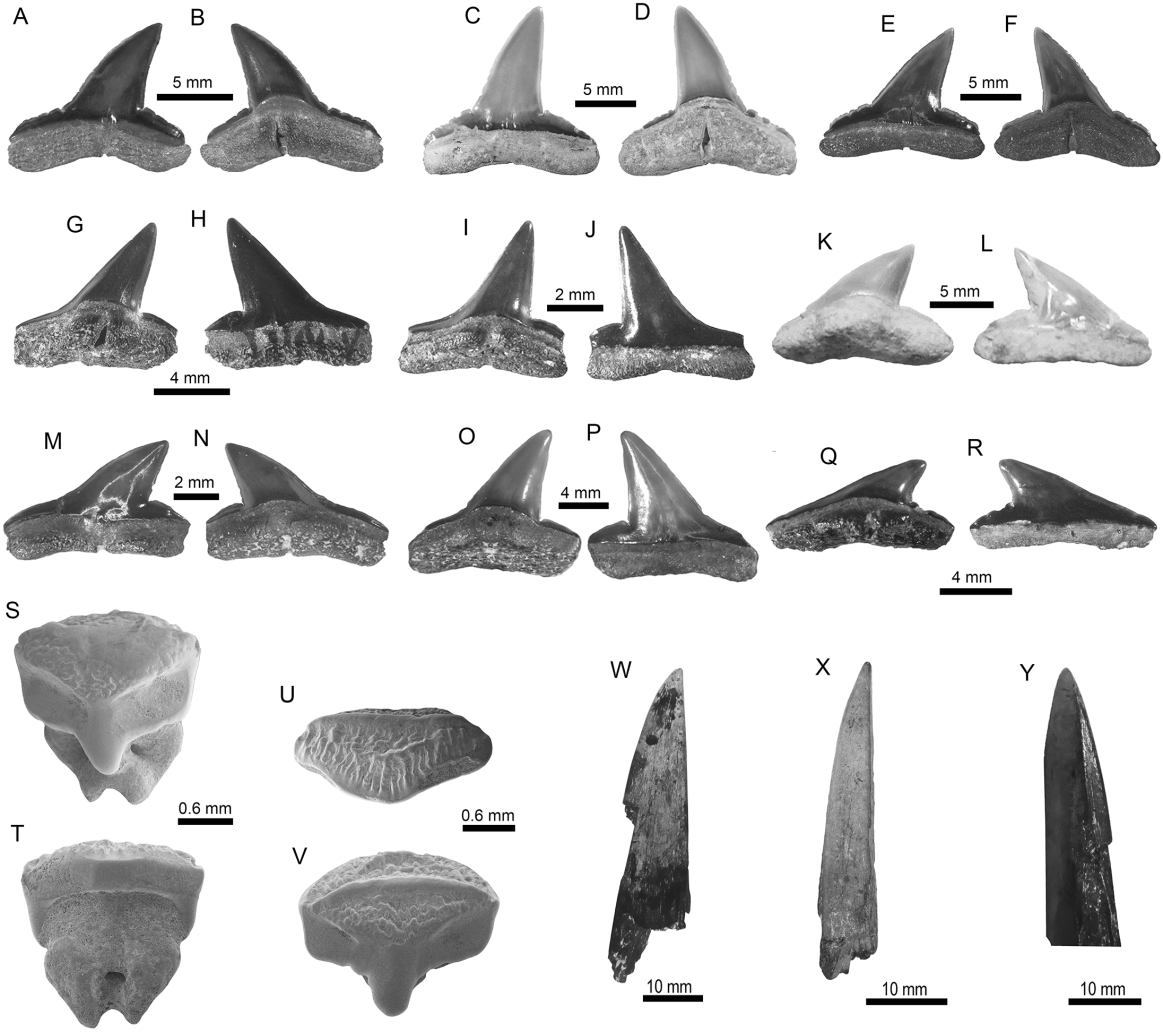
Carcharhiniformes and Rajiformes of the Pirabas Formation. A-F. †*Negaprion eurybathrodon* (A-B: MPEG-182-V; C-D: MPEG-787-V; E-F: MPEG-1542-V). G-J. †*Sphyrna arambourgi* (G-H: MPEG-1144-V; I-J: MPEG-1543-V). K-R. †*Sphyrna* cf. *S*. *laevissima* (K-L: DGM-DNPM-654-P; M-N: MPEG-1838-V; O-P: MPEG-278-V; Q-R: MPEG-811-V). S-V. *Rhynchobatus* sp. (S-T: MPEG-1951-V; U-V: MPEG-1950-V). W-Y. *Pristis* sp. (W: MPEG-1873-V; X: MPEG-1874-V; Y: MPEG-1873-V). View: labial (A, C, E, H, J, L-M, P, R, U), lingual (B, D, F-G, I, K, N-O, Q), posterior-occlusal (S), anterior-basal (T), occlusal (V), dorsal (W-Y).

**Fig 6:**
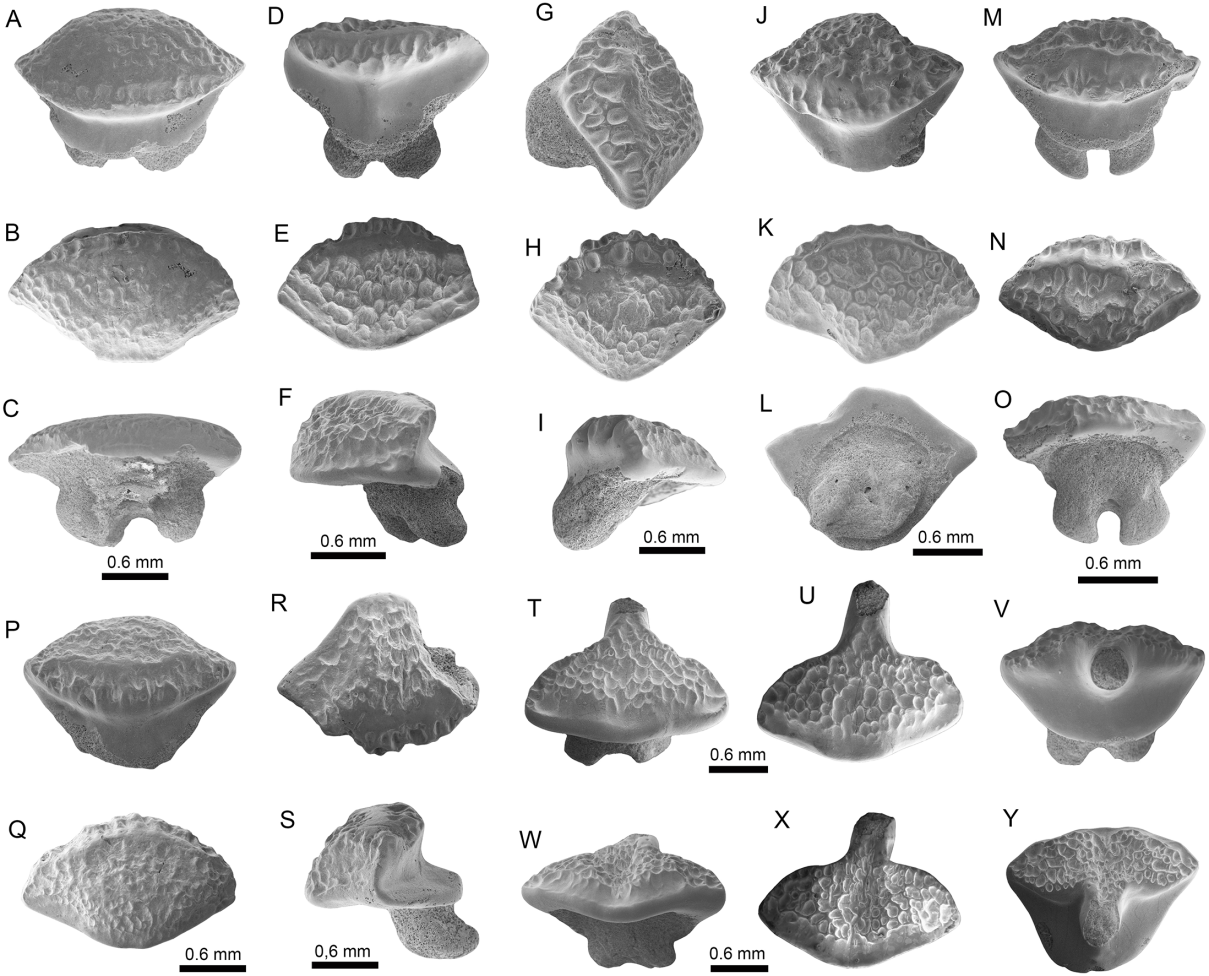
Myliobatiformes of the Pirabas Formation. A-C. cf. *Dasyatis* sp. (MPEG-1977-V). D-S. cf. *Himantura* sp. (D-F: MPEG-1959-V; G-I: MPEG-1960-V; J-L: MPEG-1968-V; M-O: MPEG-1958-V; P-Q: MPEG-1967-V; MPEG-1961-V). T-Y. *Taeniura* sp. (T-V: MPEG-1980-V; W-Y: MPEG-1982-V). View: labial (C, E, H, K, N, Q, T, X), lingual (V), basal (L), Profile (F, I, S), occlusal (A-B, D, J, M, P, R, U, Y), occlusal-profile (G), anterior-basal (O, W).

**Fig 7:**
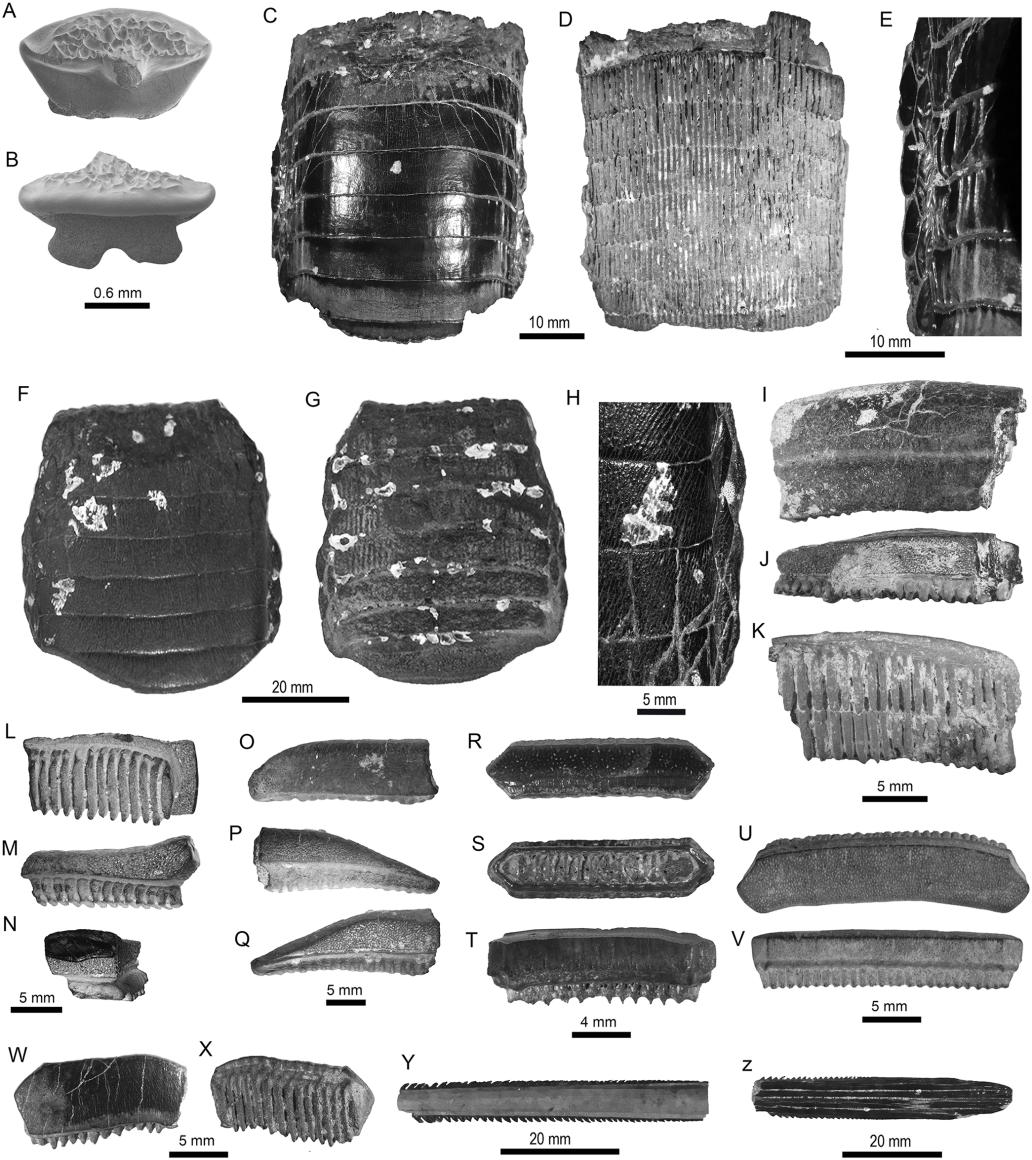
Myliobatiformes of the Pirabas Formation. A-B. *Taeniura* sp. (MPEG-1981-V). C-H. †*Aetomylaeus cubensis* (C-E: MPEG-1762-V, F-H: MPEG-1726-V). I-N. *Aetomylaeus* sp. (I-K: MPEG-1723-V; L-N: MPEG-1774-V). O-Q. Myliobatoidea Indet. (MPEG-1736-V). R-X. *Rhinoptera* sp. (R-T: MPEG-982-V; U-V: MPEG-1866-V; W-X: MPEG-1860-V). Y-Z. Myliobatiformes Indet. (Y: MPEG-1845-V; Z: MPEG-1755-V).

355 elasmobranch fossil teeth attributable to 24 taxa are identified (Figs 3–7, Table 3). The shark fauna is dominated by representatives of the Carcharhinidae Jordan and Evermann 1896 [87] (62.7% of the total assemblage), which are associated mainly with shallow water and nearshore environments. This family includes: *Galeocerdo* Müller and Henle 1837 [88] (Fig 3A–3F), *Rhizoprionodon* Whitley 1929 [89] (Fig 3G–3L), *Negaprion* Whitley 1940 [90] (Fig 5A–5F) and *Carcharhinus* Blainville 1816 [91] (Fig 3M–3Z), the latter being the most abundant taxon (Table 3).

**Table 3:** Sharks assemblages of Pirabas Formation and their Paleobiogeographic record in the Miocene.

Taxonomy Pirabas Elasmobranchs					Localities														
Superorder	Order	Family	Genus	Taxon	ARI	CAI	CPT	FOL	IDF	B5M	B17	D11	PDA	PDC	PDF	PDS	SGU	B12	Total specimens
Galeomorphii	Orectolobiformes	Hemiscyllidae	cf. *Chiloscyllium*	cf. *Chiloscyllium* sp.									1						1
		Ginglymostomatidae	*Nebrius*	*Nebrius* sp.					1		8		1	1					11
	Lamniformes	Pseudocarchariidae	*Pseudocarcharias*	*Pseudocarcharias* cf. *P*. *komoharai* (Matsubara, 1936 [92])				2			1		2						5
		†Otodontidae	†*Carcharocles*	†*Carcharocles chubutensis* (Ameghino, 1906 [93])				1		2	2		2						7
				†*Carcharocles* sp.					1	2		1	1					1	6
	Carcharhiniformes	Hemigaleidae	*Hemipristis*	†*Hemipristis serra* (Agassiz, 1835 [94])	3					1			4		5	1		1	15
		Carcharhinidae	*Galeocerdo*	†*Galeocerdo mayumbensis* (Dartevelle and Casier, 1943 [61])			1			6	13	4	1						25
			*Rhizoprionodon*	*Rhizoprionodon* sp.			1				1		3				1		6
			*Carcharhinus*	†*Carcharhinus ackermannii* (Santos and Travassos, 1960 [23])			4			1	33	3	1						42
				†*Carcharhinus gibbesii* (Woodward, 1889 [95])					1										1
				*Carcharhinus perezi* (Poey, 1876 [96])							1		2						3
				*Carcharhinus* sp.	3					20	78	21	5					1	128
			*Negaprion*	†*Negaprion eurybathrodon* (Blake, 1862 [97])		4				4	5	1	2					1	17
		Sphyrnidae	*Sphyrna*	†*Sphyrna arambourgi* (Cappetta, 1970 [64])							2								2
				†*Sphyrna* cf. *S*. *laevissima* (Cope, 1867 [98])	1	1					3		2						7
Batomorphii	Rajiformes	Rhynchobatidae	*Rhynchobatus*	*Rhynchobatus* sp.									5						5
		Pristidae	*Pristis*	*Pristis* sp.									5						5
	Myliobatiformes	Dasyatidae	cf. *Dasyatis*	cf. *Dasyatis* sp.									9						9
			cf. *Himantura*	cf. *Himantura* sp.									6						6
			*Taeniura*	*Taeniura* sp.									6						6
		Myliobatoidea	*Aetomylaeus*	†*Aetomylaeus cubensis* (Iturralde-Vinent et al. 1998 [99])							1		3						4
				*Aetomylaeus* sp.							1		20						21
		Myliobatoidea indet.	Ind.	Ind.									1						1
		Rhinopteridae	*Rhinoptera*	*Rhinoptera* sp.			2				4		14						20
	Myliobatiformes indet.	Ind.	Ind.	Ind.					2										2

Abbreviations of the listed localities (from left to right) and its respective municipalities in Pará state: ARI, Aricuru / Maracanã; CAI, Caieira / Capanema; CPT, Colônia Pedro Teixeira / Capanema; FOL, Fazenda Olaria / Capanema; IDF, Ilha de Fortaleza / São João de Pirabas; B5M, B-5 Mine / Capanema; B17, B-17 Mine / Capanema; D11, D11-Mine / Capanema; PDA, Praia de Atalaia / Salinópolis; PDC, Praia do Castelo / São João de Pirabas; PDF, Praia de Fortalezinha / Maracanã; PDS, Praia de Salinas / Salinópolis; SGU, Sítio Guilhermino / Capanema; B12, B-12 Mine, Capanema.

All related occurrences in Table 3 were based on Casier [100]; Santos and Travassos [23]; Gillette [66]; Kindlimann [101]; Kruckow and Thies [102]; Iturralde-Vinent et al. [103]; Laurito [69]; Donovan and Gunter [104]; Apolín et al. [105]; Underwood and Simon [106]; Reis [25]; Alván [107]; Laurito and Valerio [108]; Portell et al. [109]; Aguilera and Lundberg [110]; Aguilera et al. [111]; Pimiento et al. [77,78]; Carrillo-Briceño et al. [60,80–83], Southern South America [79,112–118] and North America [65,68,70,102,112,119–121], Africa, Asia and Europa [42].

Other shark families found in the Pirabas assemblage (but less abundant in relation to carcharhinids) (Table 3) include Hemiscyllidae Gill 1862 [122] (Fig 4A–4C), Ginglymostomatidae Gill 1862 [122] (Fig 4D–4G), Pseudocarchariidae Compagno 1973 [58] (Fig 4H–4K), †Otodontidae Glikman 1964 [123] (Fig 4L–4S), Hemigaleidae Hasse 1879 [124] (Fig 4T–4X), and Sphyrnidae Gill 1872 [125] (Fig 5G–5R) (Table 3). No evidence of sharks from the bathyal or meso-bathyal zone were found, except a few teeth referred to *Pseudocarcharias* Cadenat 1963 [126](Fig 4H–4K), which occur usually well offshore [127].

Concerning the batoids from the Pirabas Formation, they are characterized by Myliobatidae Bonaparte 1838 [128], Dasyatidae Jordan 1888 [129], Rhinopteridae Jordan and Evermann 1896 [87], Rhynchobatidae Garman 1913 [130], and Pristidae Bonaparte 1838 [128] taxa (Figs 5S–5Y, 6A–6Y and 7A–7Z, Table 3). With three genera, the stingrays (Dasyatidae) are the most diverse (Table 3). However, the eagle ray *Aetomylaeus* Garman 1908 [131] (Fig 7C–7N), and the cownose ray *Rhinoptera* Cuvier 1929 [132] (Fig 7R–7X), are the most abundant batoids from the assemblage (Table 3).

### Stable isotope analyses of chondrichthyan teeth

**Fig 8:**
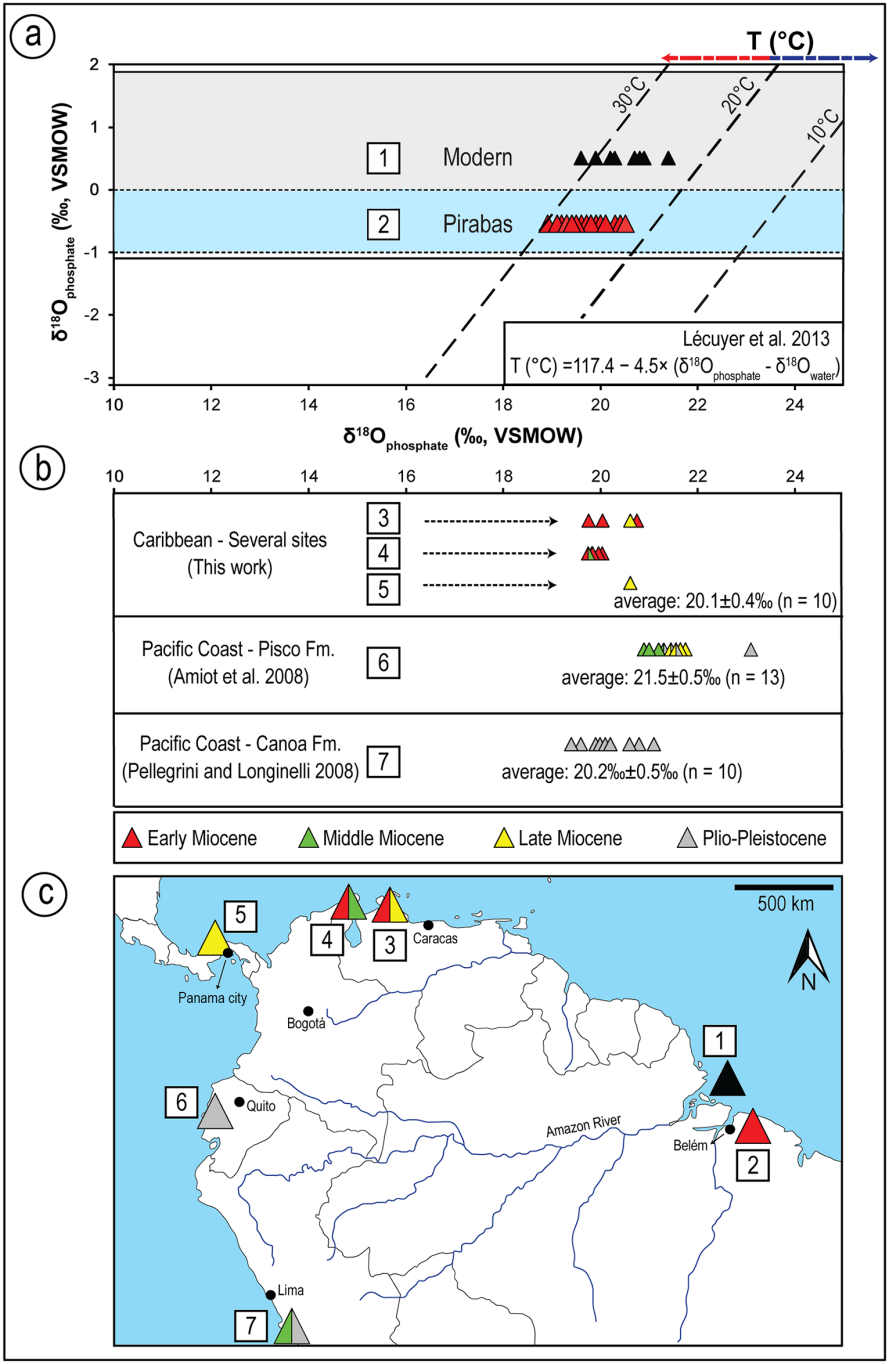
**(a)** Oxygen isotope composition of phosphate and their relationship as a function of the oxygen isotopic composition of water and temperature. Dashed lines are isotherms calculated from Lécuyer et al. [133]: The blue-shaded area surrounds the isotopic compositions measured for the Pirabbean group and represents the variation of early Miocene seawater (δ^18^Ow: -0.5 ‰, [134]); The gray-shaded zone encircles the isotopic compositions of the Recent group, characterizing the modern fluctuation in the Amazon coastal region (δ^18^Ow: 0.5 ‰, [135]; (b) Isotopic composition of shark teeth from other fossiliferous deposits of Tropical America analyzed in this work and from the literature, average δ^18^O_PO4_ values and triangle representing age; (c) Geographical map with fossiliferous units and locations that correspond to box numbers found at the side of each dataset: 1, 2 –Brazil, 3 –Venezuela, 4 –Colombia, 5 –Panama, 6 –Peru, 7 –Ecuador.

The δ^18^O_PO4_ values of the elasmobranch teeth have a range from 18.9 ‰ to 21.4 ‰ (Tables 1 and 2, Fig 8). The Pirabas fossil shark teeth have ±1.2 ‰ variation, and the values have a range between 19.3 to 19.8 ±0.4 ‰ (n = 31). The bioapatite compositions of rays have the same variation of values between 19.3 to 19.9 ±0.4 ‰ (n = 21).

Similar mean values and ranges of δ^18^O_PO4_ have been measured for fossils of †*G*. *mayumbensis* (19.6 ±0.4 ‰, n = 6), *Sphyrna* sp. (19.6 ±0.5 ‰, n = 6) and †*H*. *serra* (19.6 ±0.2 ‰, n = 6). The lowest isotope values were measured for *Carcharhinus* (19.3 ±0.3 ‰, n = 8), whereas the highest values for fossils were measured for †*C*. *chubutensis* (19.9 ±0.4 ‰, n = 5). Recent shark teeth of *C*. *leucas* from the Amazon marine platform have even higher δ^18^O_PO4_ values with a mean of 20.6 ±0.5 ‰ (n = 10).

**Fig 9:**
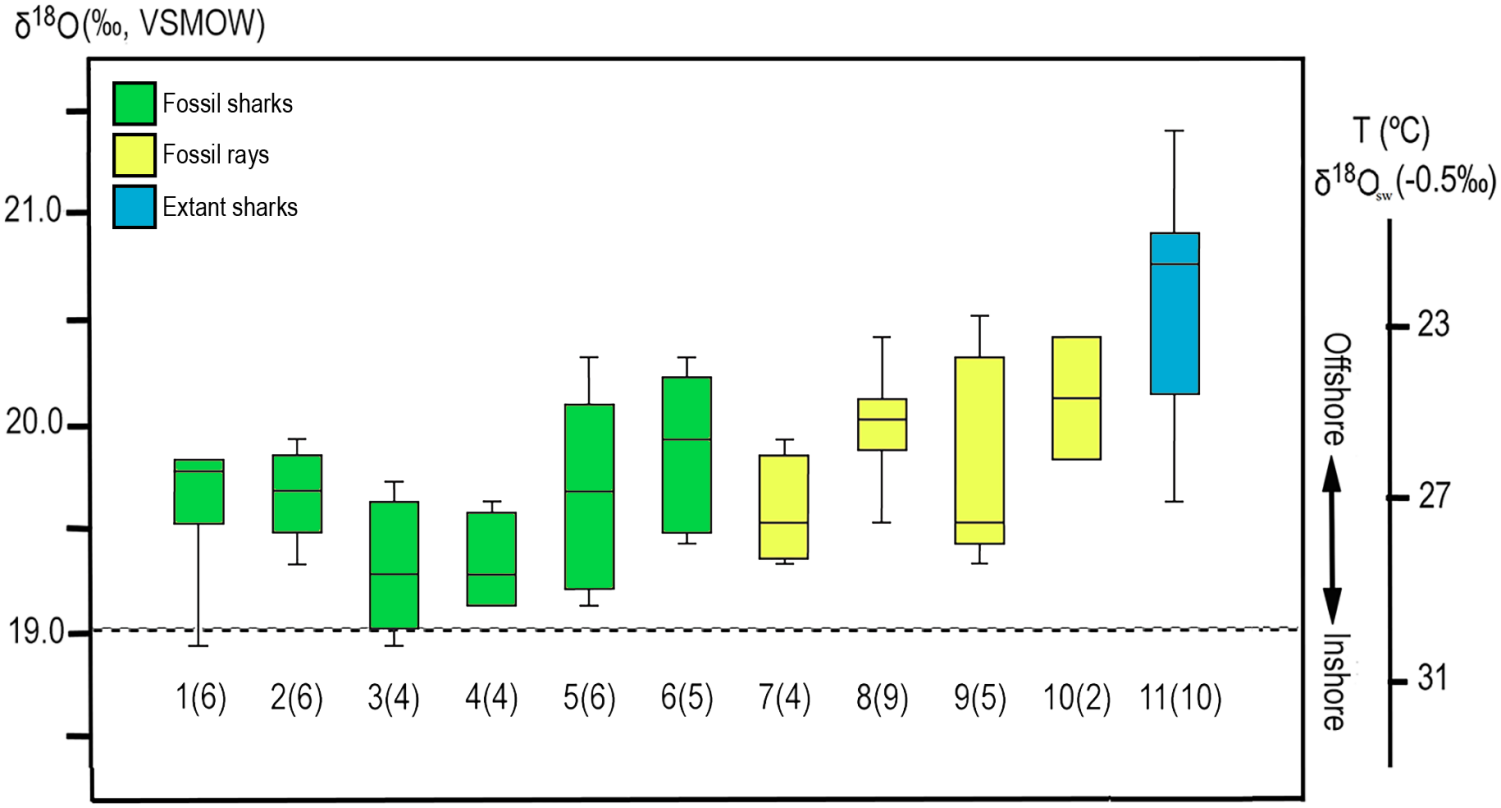
Calculated paleotemperature based on shark and ray tooth δ^18^O_PO4_ values from the Pirabas Formation. In green (left): fossil sharks, yellow (center): fossil rays, blue (right): recent sharks. Genus and **(***n*): 1. †*Galeocerdo mayumbensis* (6), 2. †*Hemipristis serra* (6), 3. *Carcharhinus* sp. (4), 4. †*Carcharhinus ackermannii* (4), 5. *Sphyrna* sp. (6), 6. †*Carcharocles chubutensis* (5), 7. †*Aetomylaeus cubensis* (4), 8. *Aetomylaeus* sp. (9), 9. Myliobatoidea (5), 10. *Rhinoptera* sp. (2), 11. *Carcharhinus leucas* (9).

The δ^18^O_PO4_ values of the batoids are slightly higher and have a similar range of variation as the sharks (19.3 ‰ to 20.5 ‰), even if only dentine was sampled (Fig 9). Tooth plates of †*A*. *cubensis* have an average δ^18^O_PO4_ value of 19.6 ± 0.3 ‰ (n = 4), while the other unassigned specimens of *Aetomylaeus* sp. have a higher mean value (20.0 ±0.3 ‰, n = 9). The latter is very similar to values of *Rhinoptera* sp. (20.1 ±0.5 ‰, n = 2; Fig 9). Other batoid teeth have more scattered values compared to the above ranges (Table 1).

Statistical tests (Student’s *t*-test, One-way ANOVA, Tukey’s pairwise) show that the three main elasmobranch datasets (fossil rays, fossil sharks and modern sharks) have significant differences between their average isotopic compositions. Fossil and extant sharks could be grouped separately as they are statistically distinct (*t*-test: t(39) = 6.48, p<0.001). Fossil rays also have a distinct average composition that sets them apart from the other groups (*t*-test: t(50) = 2.48, p<0.02). When tested within the groups for the different genera, fossil sharks and rays had no significant differences (S1 and S2 Datasets).

**Fig 10:**
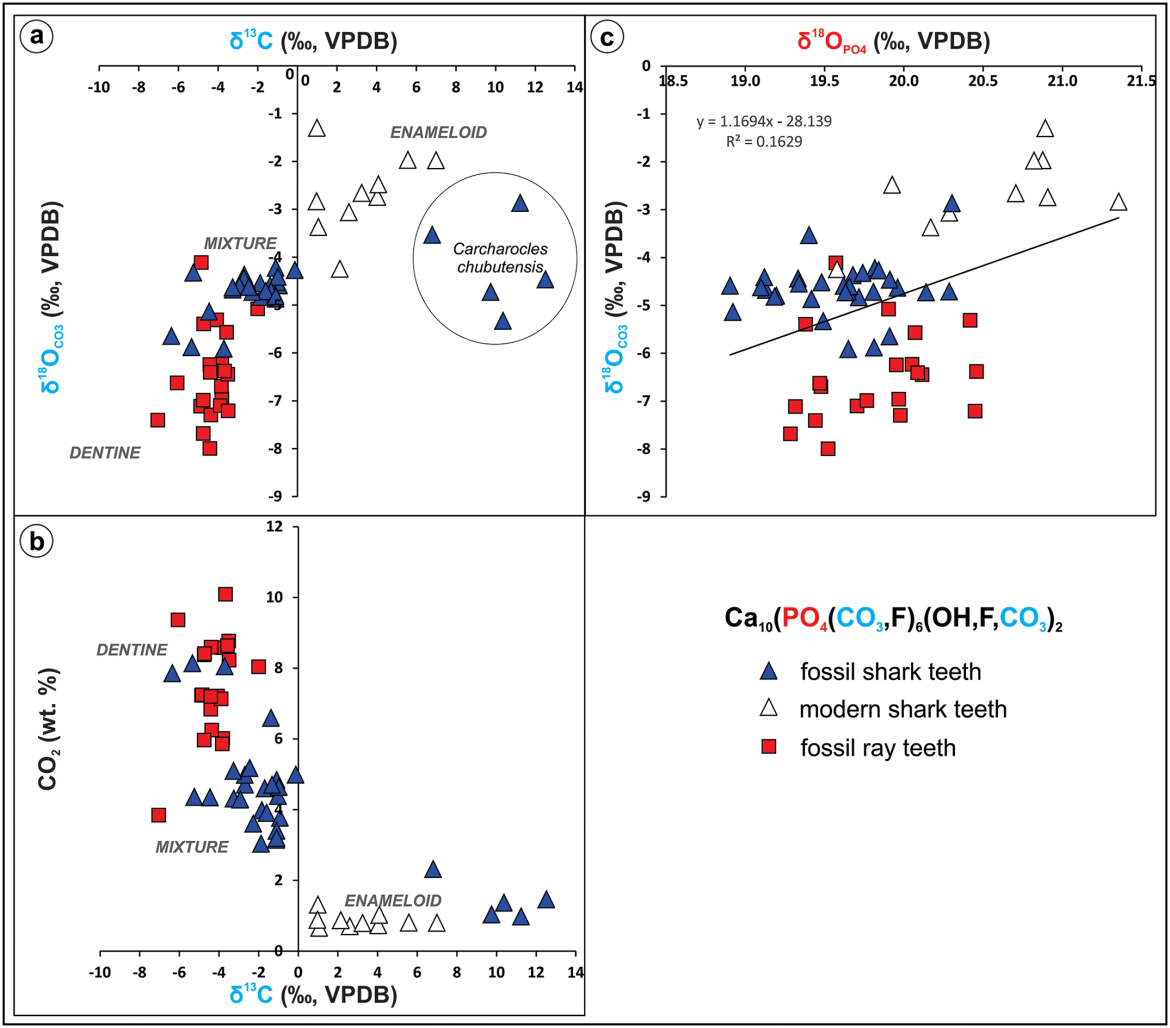
Dispersion graphs of carbonate in phosphate data. **(a)** carbon *versus* oxygen isotopic compositions; **(b)** carbonate content *versus* carbon isotopic composition: enameloid samples could be distinguished by their low carbonate content and high δ^13^C values identified in modern and fossil specimens, a similar pattern observed in previous works [29,37]; **(c)** oxygen in phosphate *versus* oxygen in carbonate isotopic compositions: no correlation could be observed between both datasets suggesting no influence from dentine remains in δ^18^O_PO4_ data.

The carbonate in phosphate (δ^13^C, δ^18^O_CO3_) isotopic compositions are different between enameloid and dentine. Samples where the enameloid could be separated (S1 Table, Fig 10) have low carbonate content (1.0 ± 0.4 wt.%, n = 15) with positive δ^13^C values from 1.0 to 12.5 ‰ and larger range in δ^18^O_CO3_ (-3.2 ‰ ± 1.1). The rays, where the enameloid is very thin or absent, along with some *Hemipristis* (HS-IV, V, VI) have isotopic compositions of dentine that are different (S1 Table, Fig 10b). The carbonate content is higher than in the enameloid (7.6 ± 1.3 wt.%, n = 24), while the values of δ^13^C (-4.4 ‰ ± 1.1) and δ^18^O_CO3_ (-6.4 ‰ ± 0.9) are lower. Other sharks’ teeth were identified with dentine and enameloid by its carbonate content of 4.4 ± 0.8 wt.% (n = 23), and their isotopic compositions are inbetween the two extremes of enameloid and dentine (δ^13^C = -2.0 ‰ ± 1.2; δ^18^O_CO3_ = -4.6 ‰ ± 0.2) (Fig 10a and 10b).

The carbonate data clearly show discrimination related to different tissues analyzed, however this cannot be said for the more resistant δ^18^O_PO4_ data as shark teeth with or without some dentine content have similar average isotopic compositions. In this regard and as observed in previous researches, tissue discrimination is a stronger factor to influence carbonate isotopic compositions in phosphate than analyzing different taxa [29,37]. Moreover, the oxygen compositions of carbonate and phosphate are not correlated (Fig 10c). Considering the consistency of δ^18^O_PO4_ values checked by statistical tests, these data are considered for further ecological and paleoenvironmental discussions.

The complementary dataset of South American sharks provided isotopic compositions slightly enriched ranging from 19.8 ‰ to 20.8 ‰ (n = 10), overlapping against the upper limit of δ^18^O_PO4_ values found for Pirabas fossil elasmobranchs. The following terms will be used in the discussion: "Pirabbean group" (Pirabas Formation elasmobranchs), "fossil shark group" (Pirabas Formation sharks), "fossil rays group" (Pirabas Formation rays) and "Recent shark group" (Recent Amazonian sharks).

## Discussion

### Faunal assemblage

Previous references to fossil elasmobranchs from the Pirabas Formation are rare (e.g. [23–26]). From the collections described here (24 shark and ray taxa) ten taxa are extinct (†*Carcharocles chubutensis*, †*Carcharocles* sp., †*Hemipristis serra*, †*Galeocerdo mayumbensis*, †*Carcharhinus ackermannii*, †*Carcharhinus gibbesii*, †*Negaprion eurybathrodon*, †*Sphyrna arambourgi*, †*Sphyrna* cf. *S*. *laevissima*, and †*Aetomylaeus cubensis*). The remaining taxa (Table 3) consist of species with living representatives in Tropical America and adjacent areas (e.g. [75,127,136]).

Some species such as cf. *Chiloscyllium* Müller and Henle 1837 [88] (Fig 3A–3C), *Nebrius* Rüppell 1837 [137] (Fig 3D–3G), and *Rhynchobatus* Müller and Henle 1837 [88] (Fig 5S–5V), which are present in our fossil fauna, only have living counterparts in the eastern Atlantic and Indo-West Pacific (e.g. [136]). The presence of cf. *Chiloscyllium* sp. in the Pirabas Formation represents the first Neogene fossil record of this taxon in the Americas, as it was previously recorded from the Upper Cretaceous of North America and Trinidad [42,138]. The presence of *Nebrius*, *Rhynchobatus* [83], and now the cf. *Chiloscyllium* in the Miocene sediments of the Americas confirms that these taxa became extinct in the Western Atlantic and Eastern Pacific, possibly as environmental changes occurred after the definitive closure of the Isthmus of Panama (e.g. [83,139–141]). With the exception of cf. *Chiloscyllium* sp., the remaining elasmobranch taxa of the Pirabas Formation has been found in other Neogene marine deposits of the Americas (e.g. [60,73,77,78,81,83]). This taxonomic commonality of the Pirabas Formation (Table 3) is better expressed by the nearby early Miocene assemblages from the Gatunian/proto Caribbean bioprovince [83].

**Fig 11:**
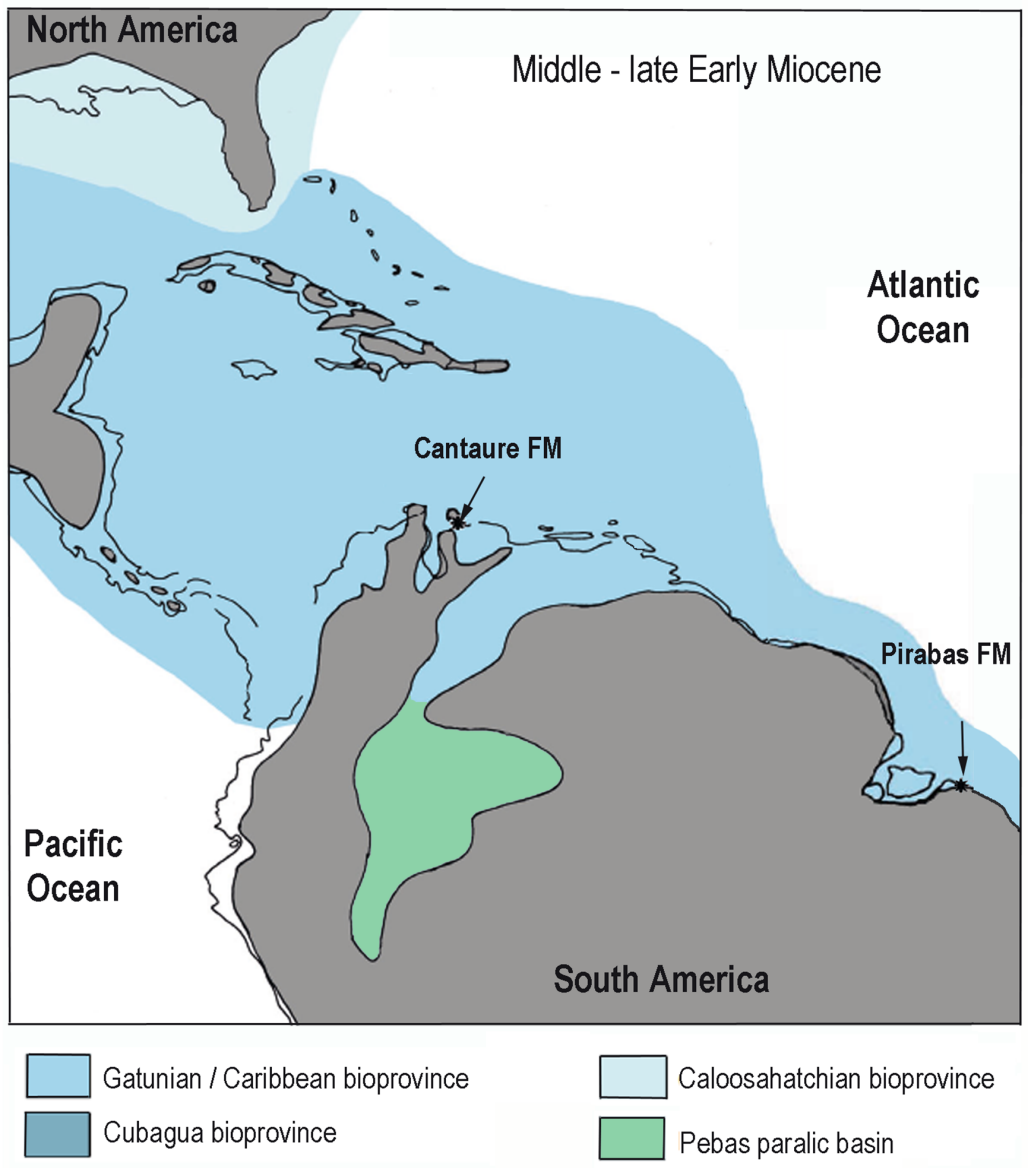
Paleogeographic range of †*Carcharhinus ackermannii* Santos and Travassos 1960 [23] from both Pirabas and Cantaure formations. Schematic reconstruction modified from [28,142–148]. Reprinted from [28,142] under a CC BY license, with permission from Aguilera O. and Schwarzhans W., original copyright 2016 and 2013 respectively (S3 Appendix).

Within the prospections realized so far in the Pirabas assemblage, †*C*. *ackermannii* and †*G*. *mayumbensis* are the most abundant shark taxa (Table 3). The fossil record for †*C*. *ackermannii* is restricted exclusively to a few full-marine early Miocene units of Brazil (Fig 11, S1 Fig) and Venezuela [83]. The fossil record of †*C*. *ackermannii* unknown in other Neogene units outside Tropical America would suggest that this species was endemic in the region during the early Miocene.

In contrast, †*G*. *mayumbensis* has been reported in the scientific literature from a few Miocene localities of Africa, Asia, North America and South America [42,61,83,149,150]. The known fossil record of †*G*. *mayumbensis* [42,61,77,83,149,150] suggests that this was a coastal-pelagic species, with a widespread distribution in tropical environments and probably restricted to the early to middle Miocene.

### Pre-Amazon delta

**Fig 12:**
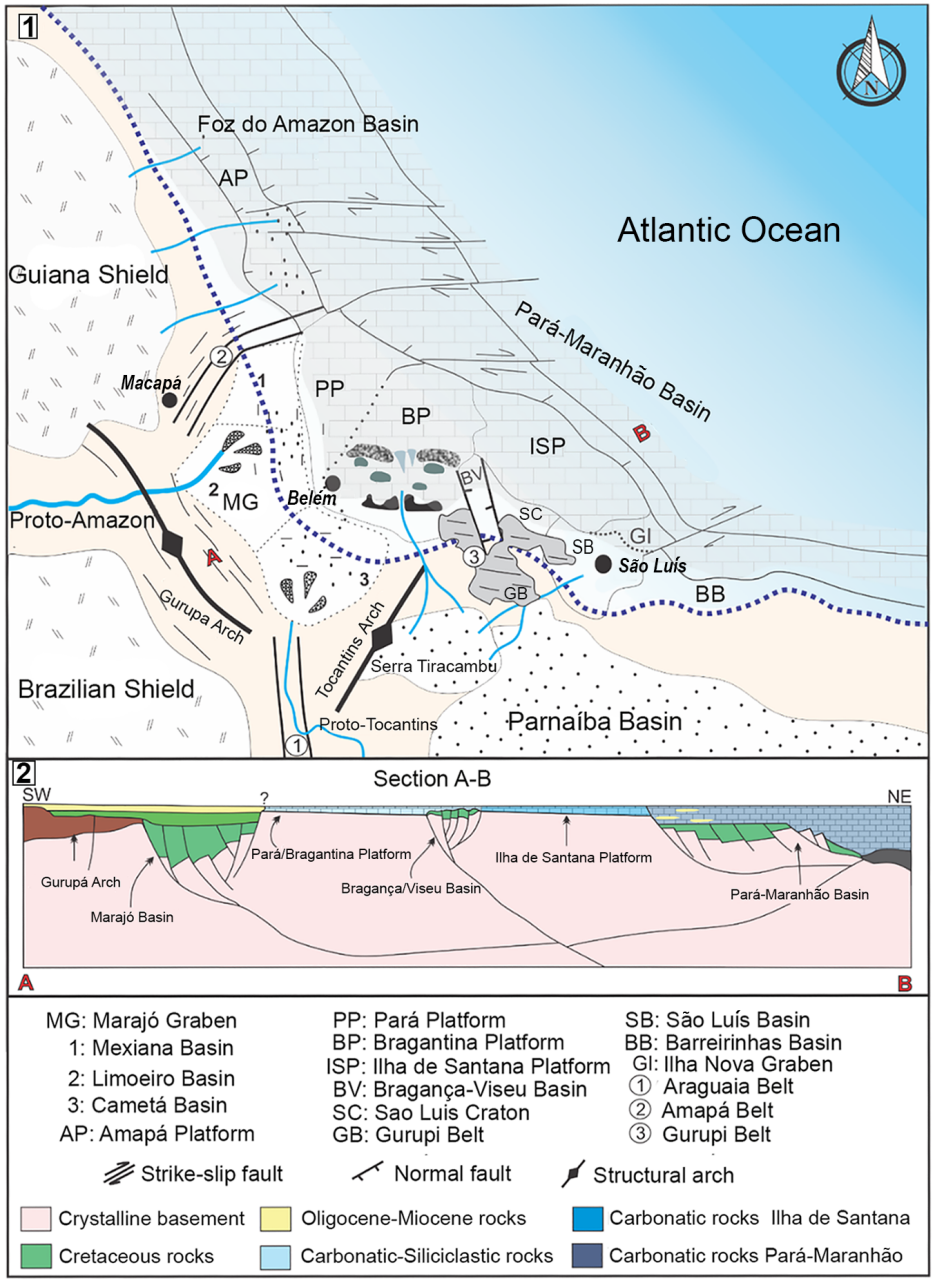
Model of the paleo-Amazon delta during the Oligocene-Miocene in the north coast of Brazil:1, regional geology and paleo-drainage systems; 2, cross section model (A-B) from the coastal plain to the marine platform.

The shallow water Oligocene-Miocene platform of North Brazil was dominated by benthic carbonate producers, such as coralline red algae, bryozoans, crinoids, echinoids, mollusks and fishes [53]. A complex of faunal assemblages of marine micro invertebrates (e. g. foraminifera and ostracods), macro invertebrates (e. g. mollusks, echinoids, crustaceans) and vertebrates (fishes, reptiles and mammals) represented an area of high productivity in rocky reef-fringing reef complexes along the North and Northeastern Atlantic coast (Fig 12). The Amazon shelf, incised by a canyon during early to late Miocene, was favorable for the paleo-Amazon fan siliciclastic deposition [15]. The first Amazon fan may have covered an area of about 330,000 km^2^ and the sediment depths accumulated may have approached 9 km [13]. Therefore, this siliciclastic input into the Atlantic coastal zone may have had a significant influence around the river mouth, causing the demise of the carbonate platform during the Plio-Pleistocene. The Pirabas carbonate platform was not exclusively affected by the first Amazon fan dynamics because the deposition area is further away from the mouth of the Amazon River. However, the siliciclastic Barreiras progradation during the middle Miocene to Pliocene (Barreiras Formation) progressively replaced the Pirabas carbonate platform [5].

### A regional stable isotope signal?

The δ^18^O_PO4_ values of elasmobranch teeth represent an instantaneous record of water parameters in which the biogenic apatite was formed. Most biological groups that synthesize phosphate biominerals have a controlled mechanism with specialized proteins capturing ions rapidly, and chemical exchange of phosphate ions is negligible through inorganic process at low temperatures [151–153]. In a pioneer study, Longinelli and Nuti [154,155] and Kolodny et al. [151] noted that δ^18^O_PO4_ values of ectothermic fishes were correlated with ambient water isotopic composition (δ^18^O_w_) and temperature. Since sharks and rays synthesize many teeth during their life, the δ^18^O_PO4_ value of each tooth should correspond to conditions of the aqueous environment where they lived at the given period of tooth formation [29]. Most shark and ray species commonly migrate at least short distances throughout their life, but even long ranging species tend to return or stay within their home areas, natal (birth) sites or other adopted localities [156–164] for extended periods prior to migration. It is possible that some of the analyzed specimens were regionally ‘Pirabbean’ given the abundance of nutrients and the presence of sheltered environments (shallow bays, river mouth regions) within the area that could support this hypothesis [27,53,55]. However, such high productive settings are also observed in fossil assemblages from adjacent regions (e. g., proto-Caribbean [60,81–83]). A larger variation in δ^18^O_PO4_ values could be expected if these selachians migrated regionally (e. g., [48,50,51]), with higher values reflecting cooler waters while lower ones recorded warmer, tropical rather than sub-tropical waters. Data from other South American localities generally have higher, more positive values compared to those studied here (Fig 8b, [50,51]). This subtropical/temperate characteristic observed in Pisco (Peru) and Canoa (Ecuador) formations could be derived both from some transient taxa used in the analyzes (e. g. *Carcharocles* relatives), but as well due to a distinct global forcing influencing the specific climatic conditions between the Pirabbean and non-Pirabbean elasmobranchs. The Pirabas setting is typical for shallow and warm water masses with very little influence of deep-cold currents [27]. Meanwhile localities that are closer to the Pacific Ocean may have been subjected to important upwelling [50], and these cooler deeper waters may have spilled over into the proto-Caribbean until the Central Panamanian Seaway (CAS) closure (Fig 11, [28,71,141,142,165]). Interoceanic (proto-Caribbean) Miocene †*H*. *serra* teeth from Venezuela (Cantaure, Caujaurao), Colombia (Jimol, Castilletes) and Panama (Chagres) deposits analyzed in parallel with Pirabbean samples again have higher δ^18^O_PO4_ values (mean: 20.1 ±0.4 ‰, n = 10, Table 1). Last but not least, inter-specific variability of δ^18^O_PO4_ values in extant specimens in South Africa [29] were up to 2.5‰, twice the values obtained here for the fossil specimens. Therefore, the δ^18^O_PO4_ values of Pirabbean samples correspond at least to a typical equatorial signal of paleoceanographic condition without upwelling influence integrated over 3 to 4 Ma.

The oxygen isotope data were converted to temperature using the equation of Lécuyer et al. [133] [T (°C) = 117.4 − 4.5× (δ^18^O_phosphate_ - δ^18^O_water_)]. Seawater isotopic composition (δ^18^O_w_) of -0.5 ‰ was used for the early Miocene samples [134] and 0.5 ‰ for the Recent samples [135]. The combined isotope and calculated temperature data are shown in Fig 8. The water column profile from the Amazon delta described by Moura et al. [166] shows nonplume and plume profiles, with consistent surface temperatures of about 28°C, and below 90 m depth about 25°C. The values obtained from the fossil and recent specimens here match well with temperatures observed in extant and fossil rays from low to mid-latitude waters [30,48,158,160,161,167]. Batoids had a slightly higher δ^18^O_PO4_ value (e. g., cooler temperature), that may be attributed to the demersal behaviour of these individuals, as recent relatives of the sampled specimens usually forage near the bottom for benthic invertebrates such as mollusks, as the most common prey in their diet [168–171]. Hence, the isotopic values of rays could reflect their ecology in inhabiting not only surface but also bottom water, with temperatures characterizing middle to lower limits of the Pirabas' waters.

Regarding the sharks, it appears that these still maintain the environmental preferences reflected in the paleontological record. Their δ^18^O_PO4_ values suggest paleotemperatures of 22°C to 32°C also noted for extant and fossil euryhaline sharks [29,33,38,48,172,173]. The higher variation present in the recent group may be attributed to the change in the regional hydrological system after the establishment of the Amazon delta fan. Karr and Showers [135] studied the oxygen isotopic composition of the open ocean Amazon shelf waters and found a large variation of up to 3 ‰ (-1 ‰ to 2 ‰) reflecting changes in seasonal runoff. As such the variations in the seawater isotope values are likely to be reflected in the δ^18^O_PO4_ values of the bioapatites [133,155]. Yet, in this study the ecosystem appears to be distinct from that of the Recent conditions. Only from the Plio-Pleistocene onwards an increased influence of the Amazon may have affected the inner shelf waters imposing a larger variation in the δ^18^O_w_ driven by also seasonal cycle [13,135]. Nevertheless, the isotherms in Fig 8 still support that the δ^18^O_PO4_ values of all the elasmobranch groups are still well characteristic of marine ecosystems.

### Ecological traits of Amazonian cartilaginous fishes based on stable isotope measurements

Although the average isotopic compositions of fossil shark are not significantly different, two end-members can be proposed, when compared pair-wised: †*C*. *chubutensis* and †*C*. *ackermannii* (*t*-test: t(7) = 2.42, p<0.045). Similarly, significantly different end-members can be recognized among the rays, on the same genus: *Aetomylaeus*. The end-members within the batoids include †*Aetomylaeus cubensis* and the other unassigned individuals of *Aetomylaeus* (*t*-test: t(11) = 2.81, p<0.016). This is possibly due to the different ecological niches (inshore *vs* offshore, Fig 9) occupied by these species. Furthermore, most genera overlap in their isotopic values indicating more generalist patterns like the tiger shark †*G*. *mayumbensis*, while others have a more specialized behavior or at least a preference to restricted niches (e. g., *Rhinoptera*). Therefore, small nuances measured in the δ^18^O_PO4_ values could be related to the ecological characteristics of the elasmobranchs.

Among the studied taxa, †*C*. *ackermannii* and †*A*. *cubensis* are probably representatives of an inshore/warmer predilection. Both have relatively low average δ^18^O_PO4_ values with low variance. It can be proposed that such sharks inhabited preferentially warm and coastal waters within a restricted habitat range, but still migrating occasionally as they also occur in other Neogene units of the Americas [42]. This behavior would be similar to extant *Carcharhinus porosus* Ranzani, 1839 [174] individuals, a small and short ranging shark very common in many coastal areas of tropical and subtropical waters in the Western Atlantic [136,175–177]. Equivalent considerations can be said about †*A*. *cubensis* species, a taxon first observed in Central America by Iturralde-Vinent et al. [99]. The four tooth plates from this group have minor differences from the *Aetomylaeus* sp. group (*n* = 9). While the former have a lower variance and also mean δ^18^O_PO4_ value, the latter group recorded a higher average δ^18^O_PO4_ value (see Fig 9). Consequently, †*A*. *cubensis* could have had a peculiar shallower-inshore behavior, while the other group probably lived in colder or deeper waters. Two hypotheses may explain why the mentioned set of samples presented divergences. The first compares different species: extant *Aetomylaeus* usually occur in nearshore waters but are also present in variable bathymetric ranges, some preferring shallower intervals (e. g. *Aetomylaeus maculatus* Gray 1834 [178]), while others may occur in offshore settings up to depths of about 150 m (e. g., *Aetomylaeus bovinus* Geoffroy Saint-Hillare 1817 [179]) [180–184]. However, it is difficult to confirm this based on isolated teeth of the unassigned specimens, and precise identification would require tooth plates similar to †*A*. *cubensis*. In contrast, it is also possible that we sampled the same species but in different stages of life. No study is available referring specifically to dentition *vs* animal size for *Aetomylaeus*, however, taking into consideration comparisons of closely related myliobatoid crushing-like teeth *vs* adult size. †*A*. *cubensis* tooth plates are very large and probably reflect adult individuals of at least 1.5 m in total length (Fig 7C–7H) [42,185,186]. The teeth of the other unspecified *Aetomylaeus* vary in sizes; generally not being as large compared to a single tooth from the plates of the other taxa and therefore could belong to smaller specimens or younger individuals (Fig 7I–7N). Given all these reasons it is possible that these larger rays were able to forage in shallower waters more frequently, being less susceptible of predation by sharks because of their size and therefore recorded lower isotopic compositions (e. g., warmer paleotemperature).

To represent the offshore predilection earlier proposed, *Carcharocles* transient shark has the highest mean value of the fossil shark group, which was expected considering the nature of the extant analogous species *Carcharodon carcharias* (or great white shark). They can occur at shallow inshore waters but are more common in the outer part of the continental shelf and remote oceanic islands. Moreover, these are one of the most wide-ranging fishes, migrating over thousands of kilometers through the ocean [177,187,188]. While migrating, long periods are spent in the pelagic habitat travelling across the ocean at depths down to about 1300 m, therefore the teeth analyzed may well have been formed in colder/deeper waters, providing higher mean δ^18^O_PO4_ values compared to other fossil shark taxa. Still, their isotopic values are within the total range of other resident selachian results (Table 1), and there is a high degree of site-fidelity in great white sharks and low interchange between populations aggregated at different coastal zones, even if their migration areas overlap for this species [177,189,190].

On that basis, we estimate that if elasmobranchian groups were not using the Pirabbean coastal waters as a protected site to give birth, the ‘Blue Amazon’ was still a valuable habitat for many species of this fishes’ group. These inferences still need further investigation using statistical tests on larger datasets and estimating species size on the available groups; nevertheless, movement patterns and ecological characteristics of sharks can be applied to understand the nature of isotopic variations [33–37,86].

## Conclusions

Taxonomic characteristics and oxygen isotope compositions of 72 teeth of sharks and rays were examined for sediments from the Pirabas Formation, Eastern Amazon, Brazil. A total of 24 taxa of sharks and rays were identified including a new fossil record for the American Neogene: cf. *Chiloscyllium* sp. Based on the phosphate bound δ^18^O_PO4_ values of biogenic apatites in many elasmobranch taxa three distinct groups were separated: a fossil shark group, a fossil ray group, and a group representing Recent sharks. Comparison between the fossil and Recent isotopic compositions led to interesting paleoecological propositions. Before the establishment of the Amazon fan, inner shelf water habitats are reflected by a smaller isotopic variation compared to the *Carcharhinus leucas* values. This divergency between isotopic compositions could be due to the coastal re-configuration with the contribution of Amazon River runoff to the Atlantic Ocean, imposing a higher outflow of ^18^O-depleted water at the river mouth. The oxygen isotope approach used allowed the ecological traits between the investigated chondrichthyans to be divided into inshore or offshore habitat preferences. This approach suggests a shallow-water predilection for †*Carcharhinus ackermannii* and †*Aetomylaeus cubensis*, species known (so far) from the Neogene of Tropical America. Further work dealing with larger datasets for recent and fossil specimens can help to refine the proposed hypotheses. Nonetheless, the information presented here underlines the importance of a multidisciplinary approach to help understand past ecological dynamics of fishes.

## Supporting information

S1 Appendix Examined specimens from the Pirabas Formation. Complete list of all chondrichthyan investigated in this study and their correspondent catalog numbers.
(DOC)

S2 Appendix Preparation of teeth samples and measuring technique. A more specific method description regarding the analytical procedures performed in this research.
(DOCX)

S3 Appendix Fig 11 permission. Authorization letter from the original authors of the Fig 11.
(DOCX)

S1 Dataset Statistical tests of sharks. One-Way ANOVA and Tukey’s pairwise multiple test of δ^18^O_PO4_ data by species using the program Past 3.08. The *p* values <0.05 indicate no significant differences.
(XLSX)

S2 Dataset Statistical tests of rays. One-Way ANOVA and Tukey’s pairwise multiple test of δ^18^O_PO4_ data by species using the Past 3.08. The *p* values <0.05 indicate no significant differences.
(XLSX)

S1 Table Carbonate in phosphate isotopic composition. δ ^13^C and δ^18^O in fossil and modern shark teeth and fossil rays tooth plate from the Pirabas Formation.
(XLSX)

S1 Fig †*Carcharhinus ackermannii* of the Pirabas Formation. A-Z. (A-B: MPEG-131-V; C-D: MPEG-988-V; E-F: MPEG-729-V; G-H: MPEG-1032-V; I: MPEG-821-V; J: MPEG-825-V; K-L: DNPM-651-P (03); M-N: DNPM-651-P; O-P: DNPM-651-P; Q-R: MPEG-827-V; S-T: MPEG-832-V; U-V: MPEG-1547-V; W-X: MPEG-1532-V; Y-Z: MPEG-1634-V). View: labial (A, C, E, G, I-K, M, O Q, S, U, W, Y), lingual (B, D, F, H, L, N, P, R, T, V, X, Z).
(TIF)
