# Supplementary material for: Neogene sharks and rays from the Brazilian ‘Blue Amazon’
Source: PLoS One. 2017 Aug 23;12(8):e0182740. doi: 10.1371/journal.pone.0182740 (PMC5568136; doi:10.1371/journal.pone.0182740)
Supplement: S1 Appendix — Complete list of all chondrichthyan investigated in this study and their correspondent catalog numbers. (DOC) [file pone.0182740.s001.doc]

**S1 Appendix. Examined specimens from the Pirabas Formation.**

cf. *Chiloscyllium* sp. MPEG-1956-V

*Nebrius* sp. MPEG- 813-V; 814-V; 1073-V; 1301-V; 1302-V; 1539-V; 1545-V; 1546-V; 1834-V; MN 2644-V; MGM-DNPM-967-P

*Pseudocarcharias* cf. *P. komoharai* (Matsubara, 1936) MPEG- 1074-V; 1851-V; 1852-V; MGM-DNPM-968-P

†*Carcharocles chubutensis* (Ameghino, 1906) MPEG- 723-V; 99-V; 224-V; 907-V; 723-V; 1548-V; MGM-DNPM-967-P.

†*Carcharocles* sp. MPEG- 97-V; 98-V; 154-V; 1733-V; 1004-V; MN-3865-V.

Isotopic analysis (CB-I to CB-V): MPEG- 1640-V; 1733-V; 98-V; 1004-V; 99-V

†*Hemipristis serra* (Agassiz, 1835) MPEG- 106-V; 108-V; 147-V; 153-V; 179-V; 273-V; 280-V; 281-V; 274-V; 725-V; 779-V; 780-V; 781-V, 782-V, 783-V; 922-V; 932-V; 938-V, 939-V; 940-V; 941-V; 1012-V; 1028-V; 1029-V; 1030-V; 1055-V; 1098-V; 1099-V; 1100-V;1101-V; 1598-V; 1599-V; 1600-V; 1748-V; 1833-V; 1866-V; 1867-V; 1868-V; 1923-V; 1924-V; 1925-V; 1926-V; 2002-V; MN 2633-V; DG UFRJ 177 pd.

Isotopic analysis (HS-I to HS-VI): MPEG- 1598-V; 1599-V; 1600-V; 1833-V; 1868-V; 2002-V

†*Galeocerdo mayumbensis* (Dartevelle and Casier, 1943) MPEG- 95-V, 96-V; 121-V; 145-V; 146-V; 149-V; 150-V; 175-V; 176-V; 180-V; 199-V; 784-V; 857-V; 861-V; 929-V; 930-V; 931-V; 943-V; 1101-V; 1102-V; 1103-V; 1274-V; 1275-V; 1299-V; 1303-V; 1549-V; 1710-V; 1742-V; 1757-V; 1854-V; 1869-V; 1905-V; MGM-DNPM-967-P; DG UFRJ 175 pd.

Isotopic analysis (GL-I to GL-VI): MPG- 121-V; 784-V; 857-V; 861-V; 1101-V; 1102-V

*Rhizoprionodon* sp. MPEG- 1590-V; 1837-V; 1929-V; 1930-V; MGM-DNPM-652-P; MGM-DNPM-653-P

†*Carcharhinus ackermannii* (Santos and Travassos, 1960) MPEG- 94-V; 112-V; 131-V; 173-V; 729-V; 786-V; 788-V; 789-V; 790-V; 791-V; 792-V; 797-V; 815-V; 816-V; 817-V; 818-V; 819-V; 820-V; 821-V; 822-V; 823-V; 824-V; 825-V; 826-V; 827-V; 832-V; 851-V; 988-V; 1131-V; 1132-V; 1142-V; 1532-V; 1533-V; 1534-V; 1535-V; 1536-V; 1538-V; 1544-V; 1547-V; 1726-V; MGM-DNPM-653-P; MGM-DNPM-651-P; DG UFRJ 176 pd; DG UFRJ 178 pd.

Isotopic analysis (CA-I to CA-IV): MPEG- 786-V; 789-V; 792-V; 823-V

†*Carcharhinus gibbesii* (Woodward, 1889) MGM-DNPM-969-P

*Carcharhinus perezi* (Poey, 1876) MPEG- 1836-V, 1541-V

*Carcharhinus* spp. MPEG- 93-V; 94-V; 100-V; 101-V; 102-V; 104-V; 109-V; 110-V; 113-V; 114-V; 117-V; 119-V; 127-V; 130-V; 133-V; 155-V; 156-V; 157-V; 158-V; 159-V; 160-V; 162-V; 163-V; 164-V; 165-V; 167-V; 168-V; 170-V; 173-V; 178-V; 180-V; 183-V; 184-V; 186-V; 189-V; 190-V; 191-V; 192-V; 193-V; 194-V; 195-V; 198-V; 274-V; 275-V; 276-V; 277-V; 726-V; 727-V; 730-V; 771-V; 772-V; 773-V; 771-V; 785-V; 795-V; 796-V; 828-V; 829-V; 830-V; 831-V; 833-V; 834-V; 835-V; 842-V; 848-V; 849-V; 850-V; 859-V; 860-V; 906-V; 919-V; 920-V; 921-V; 933-V; 934-V; 935-V; 936-V; 937-V; 950-V; 951V; 952-V; 953-V; 954-V; 975-V; 977-V; 978-V; 979-V; 978-V; 1013-V; 1031-V; 1032-V; 1033-V; 1034-V; 1035-V; 1036-V; 1064-V; 1081-V; 1082-V; 1083-V; 1084-V; 1085-V; 1086-V; 1087-V; 1106-V; 1107-V; 1108-V; 1109-V; 1113-V; 1140-V; 1141-V; 1153-V; 1154-V; 1155-V; 1156-V; 1266-V; 1267-V; 1296-V; 1297-V; 1298-V; 1300-V; 1311-V; 1312-V; 1313-V; 1333-V; 1339-V; 1340-V; 1341-V; 1342-V; 1343-V; 1344-V; 1345-V; 1346-V; 1355-V; 1716-V; 1747-V; 1853-V; 1897-V; 1927-V; 1928-V; 1949-V.

Isotopic analysis (CP-I to CP-IV): MPEG 101-V; 102-V; 162-V; 1082-V

†*Negaprion eurybathrodon* (Blake, 1862) MPEG- 122-V; 175-V; 182-V; 195-V; 197-V; 787-V; 1008-V; 1540-V; 1542-V; 1582-V; 1550-V; 1751-V; 1896-V; DGM-DNPM-654-P

†*Sphyrna arambourgi* (Cappetta, 1970) MPEG- 144-V; 1543-V.

†*Sphyrna* cf. *S. laevissima* (Cope, 1867) MPEG- 278-V; 811-V; 1537-V; 1838-V; 1151-V; 1870-V; DGM-DNPM-654-P; DG UFRJ 174 pd.

Isotopic analysis (*Sphyrna* sp., SM-I to SM-VI): MPEG- 778-V; 843-V; 987-V; 1059-V; 1068-V; 1069-V

*Rhynchobatus* sp. MPEG- 1950-V; 1951-V; 1952-V; 1953-V; 1954-V; 1955-V

*Pristis* sp. MPEG- 1764-V; 1835-V; 1873-V; 1874-V; 1995-V.

Isotopic analysis (PT-I): MPEG- 1874-V

cf. *Dasyatis* sp. MPEG- 1967-V; 1968-V; 1970-V; 1977-V; 1978-V; 1979-V; 1987-V; 1990-V; 1992-V

cf. *Himantura* sp. MPEG- 1958-V; 1959-V; 1960-V; 1961-V; 1962-V; 1969-V

*Taeniura* sp. MPEG- 1980-V; 1981-V; 1982-V; 1988-V; 1989-V; 1991-V

†*Aetomylaeus cubensis* (Iturralde-Vinent et al. 1998) MPEG- 1521-V; 1522-V; 1726-V; 1762-V.

Isotopic analysis (AC-I to AC-IV): MPEG- 1762-V; 1726-V; 1521-V; 1522-V

*Aetomylaeus* sp. MPEG- 904-V; 1061-V; 1709-V; 1723-V; 1724-V; 1729-V; 1730-V; 1738-V; 1743-V; 1744-V; 1745-V; 1749-V; 1750-V; 1761-V; 1768-V; 1771-V; 1773-V; 1774-V; 1861-V; 1862-V; 1863-V; 1872-V; 1883-V; 1892-V; 1907-V; 1908-V; 1871-V; 1875-V; MN 2645-V.

Isotopic analysis (AE-I to AE-IX): MPEG- 1729-V; 1907-V; 1892-V; 1872-V; 1761-V; 1883-V; 1861-V; 1862-V; 1908-V

Myliobatoidea indet. MPEG- 1704-V; 1705-V; 1736-V; 1844-V; 1855-V.

Isotopic analysis (MY-I to MY-V): MPEG- 1704-V; 1705-V; 1844-V (3 spec. catalog, 2 analyzed); 1855-V

*Rhinoptera* sp. MPEG- 734-V; 876-V; 877-V; 982-V; 1703-V; 1711-V; 1735-V; 1739-V; 1740-V; 1741-V; 1759-V; 1769-V; 1839-V; 1844-V; 1866-V; 1898-V; 1993-V; 1994-V; MGM-DNPM-658-P; DG UFRJ 179 pd.

Isotopic analysis (RH-I, RH-II): MPEG- 1844-V; 1703-V

Myliobatiformes Ind. MPEG- 1734; 1755; 1845; MN 2640-V; MN 3866-V

*Carcharhinus leucas* (Muller and Henle 1839) Isotopic analysis, MPEG- Paleontological collection (CL-I to CL-II): CO-342; CO-343. MPEG- Ichthyologic collection (CL-III): 4475-V; (CL-IV to CL-V): reference material without catalog n.
