# Supplementary material for: Neogene sharks and rays from the Brazilian ‘Blue Amazon’
Source: PLoS One. 2017 Aug 23;12(8):e0182740. doi: 10.1371/journal.pone.0182740 (PMC5568136; doi:10.1371/journal.pone.0182740)
Supplement: S2 Appendix — A more specific method description regarding the analytical procedures performed in this research. (DOCX) [file pone.0182740.s002.docx]

**S2 Appendix. Preparation of Teeth Samples and Stable Isotope Measuring Technique.**

The samples were powdered and homogenized in an agate mortar and pre-treated following the chemical procedure of Koch et al. [1]. NBS-120c phosphorite reference material was prepared in parallel with each batch. After this step the oxygen and carbon isotopic compositions of structural carbonate in phosphate (δ^18^O_CO3_, δ^13^C) were directly analyzed on the pre-cleaned sample powders using a Gasbench II coupled to a Finnigan MAT Delta Plus XL mass spectrometer. The measured isotopic ratios were normalized to an in-house Carrara marble calcite standard calibrated against NBS-19. The analytical precision for this method is better than ±0.1‰ for O and C isotopes [2]. The isotopic values are expressed in δ-notation relative to VPDB (Vienna Pee Dee Belemnite). To obtain the phosphate oxygen isotopic composition (δ^18^O_PO4_), a silver phosphate precipitation method modified after O'Neil et al. [3] and Dettman et al. [4], described in detail by Kocsis [5] was applied. The δ^18^O_PO4_ was analyzed on a TC/EA (high-temperature conversion elemental analyzer) [6] coupled to a Finnigan MAT Delta Plus XL mass spectrometer, where the silver phosphate is converted to CO at 1450 °C via reduction with graphite. The results were corrected to in-house Ag_3_PO_4_ phosphate standards (LK-2 L: 12.1‰ and LK-3 L: 17.9‰) that had better than ±0.3‰ (1σ) standard deviations during measurements. For the NBS-120c phosphorite reference material an average value of 21.4‰ ±0.1‰ (n = 6) was obtained. The isotope ratios are expressed in the δ-notation relative to Vienna Standard Mean Ocean Water (VSMOW).

**References**

1. Koch PPL, Tuross N, Fogel ML. The effects of sample treatment and diagenesis on the isotopic integrity of carbonate in biogenic hydroxylapatite. J Archaeol Sci. 1997;24: 417–429. doi:10.1006/jasc.1996.0126

2. Spötl C, Vennemann TW. Continuous-flow isotope ratio mass spectrometric analysis of carbonate minerals. Rapid Commun Mass Spectrom. 2003;17: 1004–1006. doi:10.1002/rcm.1010

3. O’Neil JR, Roe L, Reinhard E, Blake R. A rapid and precise method of oxygen isotope analysis of biogenic phosphate. Isr J Earth Sci. 1994;43: 203–212.

4. Dettman DL, Kohn MJ, Quade J, Ryerson FJ, Ojha TP, Hamidullah S. Seasonal stable isotope evidence for a strong Asian monsoon throughout the past 10.7 m.y. Geology. 2001;29: 31. doi:10.1130/0091-7613(2001)029<0031:SSIEFA>2.0.CO;2

5. Kocsis L. Geochemical compositions of marine fossils as proxies for reconstructing ancient environmental conditions. Chimia. 2011;65: 787–791. doi:10.2533/chimia.2011.787

6. Vennemann TW, Fricke HC, Blake RE, O’Neil JR, Colman A. Oxygen isotope analysis of phosphates: a comparison of techniques for analysis of Ag_3_PO_4_. Chem Geol. 2002;185: 321–336. doi:10.1016/S0009-2541(01)00413-2
