# Supplementary figures and images for: Neogene sharks and rays from the Brazilian ‘Blue Amazon’
Source: PLoS One. 2017 Aug 23;12(8):e0182740. doi: 10.1371/journal.pone.0182740 (PMC5568136; doi:10.1371/journal.pone.0182740)

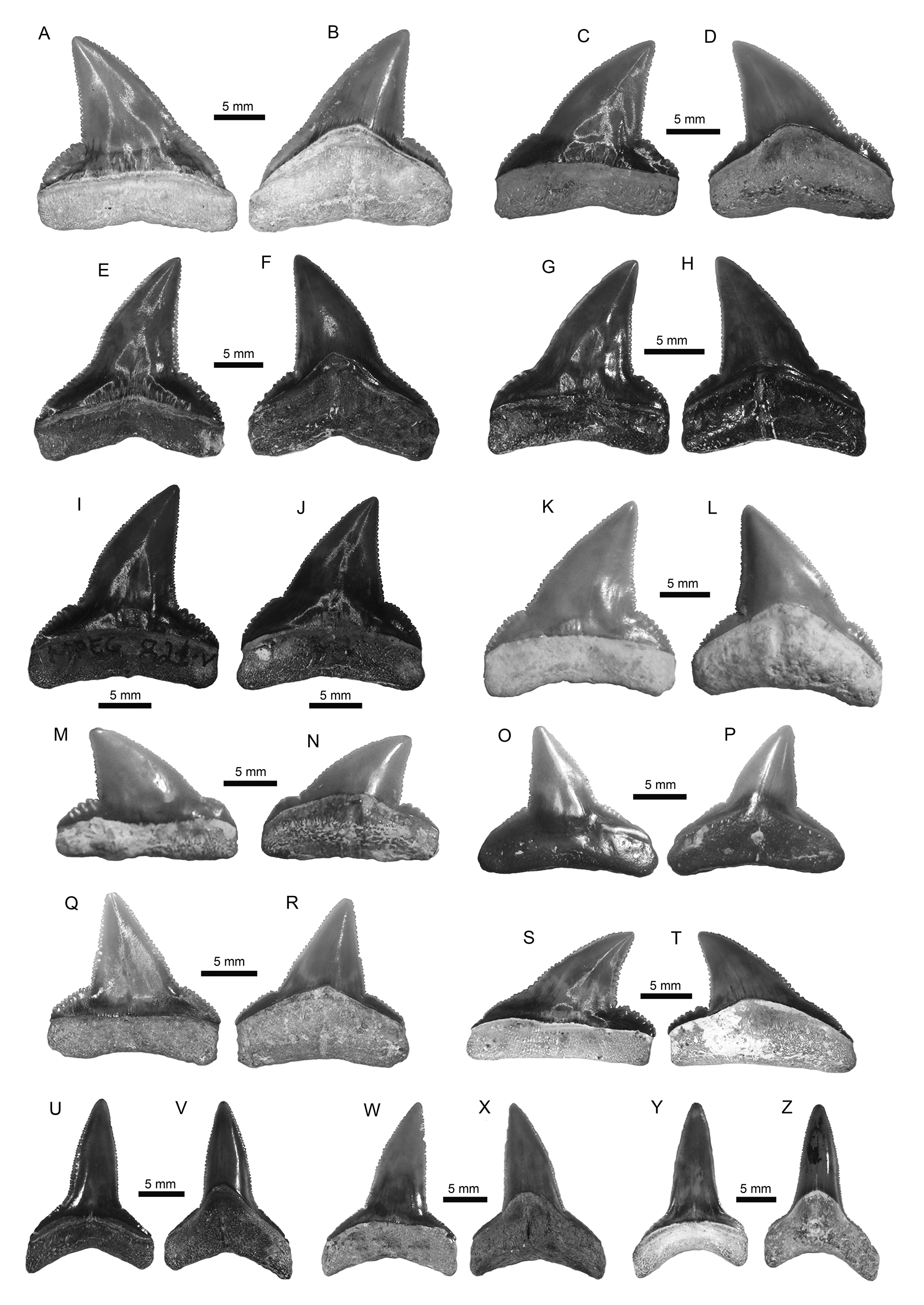

Supplement: S1 Fig — A-Z. (A-B: MPEG-131-V; C-D: MPEG-988-V; E-F: MPEG-729-V; G-H: MPEG-1032-V; I: MPEG-821-V; J: MPEG-825-V; K-L: DNPM-651-P (03); M-N: DNPM-651-P; O-P: DNPM-651-P; Q-R: MPEG-827-V; S-T: MPEG-832-V; U-V: MPEG-1547-V; W-X: MPEG-1532-V; Y-Z: MPEG-1634-V). View: labial (A, C, E, G, I-K, M, O Q, S, U, W, Y), lingual (B, D, F, H, L, N, P, R, T, V, X, Z). (TIF) [file pone.0182740.s007.tif]
