# Supplementary material for: Neogene sharks and rays from the Brazilian ‘Blue Amazon’
Source: PLoS One. 2017 Aug 23;12(8):e0182740. doi: 10.1371/journal.pone.0182740 (PMC5568136; doi:10.1371/journal.pone.0182740)
Supplement: S3 Appendix — Authorization letter from the original authors of the Fig 11. (DOCX) [file pone.0182740.s003.docx]

Lausanne, April 21, 2017

Dear Editor,

I confirm that me and Werner Schwarzhans grant permission for the open-access journal PLOS ONE to publish the Figure 11 in the research article entitled *Neogene sharks and rays from the Brazilian ‘Blue Amazon’* under the Creative Commons Attribution License (CCAL) CC BY 4.0.

Below goes the message sent by Werner Schwarzhans about the subject:

“**De:** Werner Schwarzhans <[wwschwarz@aol.com](mailto:wwschwarz@aol.com)>
**Data:** 20 de abril de 2017 12:35:43 BRT
**Para:** Orangel Aguilera <[orangel.aguilera@gmail.com](mailto:orangel.aguilera@gmail.com)>
**Assunto:** **Re: Copyright PlosOne**
**Responder A:** [wwschwarz@aol.com](mailto:wwschwarz@aol.com)

Dear Orangel,

Your figure 11 is properly referenced in the captions and its original has been published in an open source fashion with Verlag Pfeil. Therefore, we still own the copyrights for it. The only other person you have to ask is me, and I agree.
Good success,
Werner”

If additional information is needed authors are available to provide (contacts below)

With our best regards,

Orangel Antonio Aguilera Socorro

First author, e-mail: <orangel.aguilera@gmail.com>

Current address: Universidade Federal Fluminense

Departamento de Biologia Marinha, Instituto de Biologia

Niterói, Rio de Janeiro, Brasil

Werner Schwarzhans, e-mail: [wwschwarz@aol.com](mailto:wwschwarz@aol.com)

University of Copenhagen

Natural History Museum of Denmark

Copenhagen, Denmark
